# Renal Health Through Medicine–Food Homology: A Comprehensive Review of Botanical Micronutrients and Their Mechanisms

**DOI:** 10.3390/nu16203530

**Published:** 2024-10-18

**Authors:** Yi Zhao, Jian-Ye Song, Ru Feng, Jia-Chun Hu, Hui Xu, Meng-Liang Ye, Jian-Dong Jiang, Li-Meng Chen, Yan Wang

**Affiliations:** 1 State Key Laboratory of Bioactive Substance and Function of Natural Medicines, Institute of Materia Medica, Chinese Academy of Medical Sciences and Peking Union Medical College, Beijing 100050, China; 2 Department of Nephrology, State Key Laboratory of Complex Severe and Rare Diseases, Peking Union Medical College Hospital, Chinese Academy of Medical Science and Peking Union Medical College, Beijing 100730, China

**Keywords:** botanicals, medicine–food homology, kidney protection, gut–kidney axis

## Abstract

Background: As an ancient concept and practice, “food as medicine” or “medicine–food homology” is receiving more and more attention these days. It is a tradition in many regions to intake medicinal herbal food for potential health benefits to various organs and systems including the kidney. Kidney diseases usually lack targeted therapy and face irreversible loss of function, leading to dialysis dependence. As the most important organ for endogenous metabolite and exogenous nutrient excretion, the status of the kidney could be closely related to daily diet. Therefore, medicinal herbal food rich in antioxidative, anti-inflammation micronutrients are ideal supplements for kidney protection. Recent studies have also discovered its impact on the “gut–kidney” axis. Methods: Here, we review and highlight the kidney-protective effects of botanicals with medicine–food homology including the most frequently used *Astragalus membranaceus* and *Angelica sinensis* (Oliv.) Diels, concerning their micronutrients and mechanism, offering a basis and perspective for utilizing and exploring the key substances in medicinal herbal food to protect the kidney. Results: The index for medicine–food homology in China contains mostly botanicals while many of them are also consumed by people in other regions. Micronutrients including flavonoids, polysaccharides and others present powerful activities towards renal diseases. Conclusions: Botanicals with medicine–food homology are widely speeded over multiple regions and incorporating these natural compounds into dietary habits or as supplements shows promising future for renal health.

## 1. Introduction

The kidneys are indispensable for human physiological functions, including the excretion of drugs and nutrients. The kidneys maintain the balance of water, electrolytes, and endogenous metabolites throughout the body through the processes of glomerular filtration, tubular reabsorption, and tubular secretion. Additionally, they serve as the predominant organ responsible for the excretion of exogenous substances, including drugs and food nutrients [1]. However, this feature also renders the kidneys highly susceptible to acute or chronic injuries caused by these exogenous substances [2]. For example, drug-induced nephrotoxicity (DIN) accounts for up to 60% of cases of acute kidney injury (AKI) and the correlation increases in elder patients [1,3]. Dietary-resourced substances could also lead to abnormal renal status, for instance, oxalate and oxalate precursors as key determinants of urinary oxalate excretion and stone disease. In addition to certain substances that induce injury, the kidney is also affected by metabolic disorders (e.g., diabetic kidney disease) and immune abnormalities (e.g., membranous glomerulonephritis and immunoglobulin A nephropathy). The underlying molecular mechanism of kidney disorders is a complex phenomenon involving oxidative stress, inflammation, fibrosis, and mitochondrial dysfunction. Recently, the vital role of the “gut–kidney” axis, which is closely related to daily diet, has been identified and investigated [4]. However, contemporary renal disease therapy is predominantly supportive, comprising diuretics, renin–angiotensin–aldosterone system inhibitors, and immunosuppressants with a paucity of targeted pharmaceutical agents [2]. Meanwhile, these treatments could hardly reverse the continuous decline in renal function and the formation of end-stage renal disease (ESRD) [5]. Compared to drug treatment, certain daily diet patterns may also have an impact on renal function [6,7]. Natural food plants are extremely wealthy in nutrients and biological active compounds worth exploring and utilizing. Therefore, there is a promising future to explore botanicals with medicine–food homology to alleviate or prevent kidney disease.

In fact, the concept of food as medicine, or “Yao Shi Tong Yuan” in Chinese, is a fundamental tenet of Traditional Chinese Medicine (TCM). It underscores the dual function of specific foods in offering both nutritional and therapeutic advantages. The use of herbal food for medical purposes has a long and illustrious history in numerous other regions, including Japan, Korea, South America, and Europe. Despite the complexity of its ingredients, preparation methods, and active components, medicinal herbal food has been shown to have significant effects on a number of disorders, including diabetes mellitus, allergic diseases, pancreatitis, depression, and renal disease [8,9,10,11]. In the context of renal disease, a considerable number of botanical foods have been observed to exert a protective effect, as evidenced by a reduction in serum creatinine, blood urea nitrogen, histopathological injury, and fibrosis degree [12,13,14]. Early in 2002 [15], the National Health Commission (NHC) of the People‘s Republic of China had published the list of natural medical food including mostly different kinds of herbal ones. Being up to date, the list has expanded to 102 kinds of natural food including *Astragalus membranaceus*, *Codonopsis tangshen* Oliv., and *Poria cocos* (Schw.) Wolf. These herbal foods are frequently consumed by Chinese people either for their flavors or their potential health benefits. Many of them had also demonstrated renal-protective effects through anti-inflammation [16], antioxidation [17,18], and regulating mitochondria [19] and gut microbiota [20] with various micronutrients in them. The Food and Drug Administration (FDA) of the US have also established guidelines for the safety and efficacy of dietary supplements for medical use [21]. The 2020–2030 strategic plan for nutrition research by National Institutes of Health (NIH) had even placed “food as medicine” as one of the strategic goals [22], indicating the potential of medicine–food homology.

**Figure 1:**
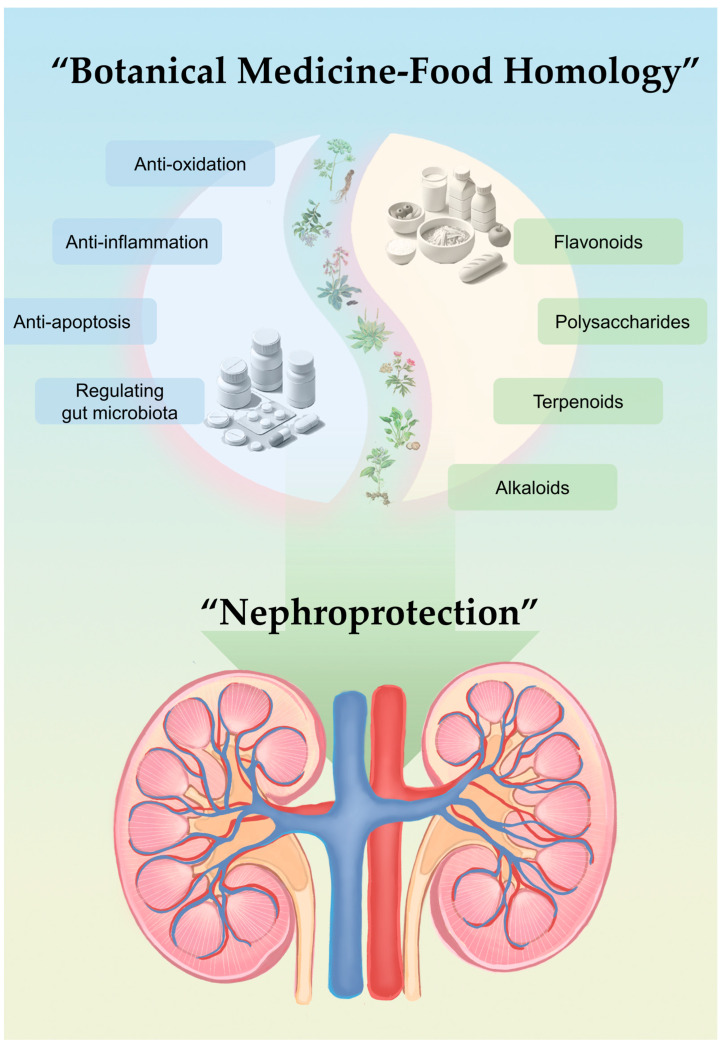
Illustration abstract drawn by authors.

Here, we reviewed the frequently used medicinal herbal food in China, the US, and Europe, and summarized the potential renal-protective effects and mechanism of these medicinal herbal foods as shown in Figure 1, providing a better basis for utilizing and exploring the key substances in botanicals with medicine–food homology.

## 2. Botanical Ingredients with Medicine–Food Homology in China, the US, and Europe

The use of botanical ingredients for dietary supplements or even for food is a widespread tradition around the world. In addition to China, the popularity of botanical dietary supplements is also evident in various European countries. Dietary supplements represent 15–20% of the total botanical market [23]. However, the complex compounds present in botanical ingredients also pose a potential risk. One of the most illustrative examples of a botanical-induced health risk is the aristolochic acid nephropathy (AAN) incident that occurred in the early 1990s [24]. The ingestion of aristolochic acids present in certain herbs had resulted in the development of AAN in numerous patients, leading to chronic renal failure and ESRD in Belgium, China, and Korea [24,25,26]. Considering the widespread and covert use of herbs, some researchers estimated that the AAN was still underestimated and called for a stronger and systematic supervision system of herbal medicine or ingredients [25]. To ensure the safety and efficiency of botanical ingredients and supplements, many regulations and standards have been published to restrict the addition of botanical ingredients in dietary supplements. In 2002, the NHC of China had published the first index of ingredients that could be both food and medication [15]. The initial version contained 92 unique edible parts including mostly botanical ingredients. Many of them have long been thought to have kidney-protective effects like *Astragalus membranaceus* and *Lycium barbarum* L. [16,27,28]. In recent years, there has been a growing interest in botanical dietary supplements, leading the NHC to edit the index in 2019 [29] and 2023 [30]. This resulted in the addition of 15 new botanical ingredients. In 2021, the NHC had also published related regulations for this index including the criteria for botanical ingredients as both food and medication [31]. The fundamental criteria consist of the following requirements: (i) have been traditionally consumed as food; (ii) have been listed in the *Pharmacopoeia* of the PRC; (iii) no food safety problems have been found in the safety assessment; (iv) comply with relevant laws and regulations on the protection of Chinese herbal medicines, wild animals, plants, and ecological resources. For the first rule, it also requires evidence for the ingredients to have been consumed as food for more than 30 years. Concurrently, all of these ingredients are also included in the *Pharmacopoeia* of the People’s Republic of China, which demonstrates their efficacy as medicinal agents. In addition to botanicals that are regarded as both food and medicine, the NHC had also delineated a list of medicinal herbs that may be included in dietary supplements (Table S1) [15]. With regard to the European countries, the regulation of botanical ingredients is also divided into two distinct categories: medicine and food supplements. The European Medicines Agency (EMA) bears the responsibility of assessing the safety and efficacy of herbal preparations when utilized as medicines, while the European Food Safety Authority (EFSA) is tasked with the oversight of herbal preparations employed as food supplements. The evaluation of botanicals used in food products was initiated in 2004, when the EFSA mandated its Scientific Committee to develop a science-based toolkit for the safety assessment of botanicals and botanical preparations [32]. Subsequently, in 2009, the EFSA published the inaugural edition of the Compendium of Botanicals, which provided information regarding the safety risks associated with botanical ingredients in dietary supplements [32]. In the United States, the Dietary Supplement Health and Education Act (DSHEA) of 1994 initially delineated the status of herbs in dietary supplements, alongside that of vitamins, minerals, amino acids, and other substances [33]. While there is no comprehensive list of ingredients used in dietary supplements by the FDA, the NIH has established a Dietary Supplement Label Database (DSLD) [34] that includes as many of these supplements as possible. 

Table 1 shows a full list of botanicals in the China index for botanicals with medicine–food homology. It contains a total of 91 botanicals, the majority of which are also included in dietary supplements in the United States and the European Union, as indicated by the EFSA Compendium of Botanicals and the DSLD. However, only a limited number of the listed botanicals are regarded as herbal medicines in accordance with the EMA standard. This is a markedly different situation from that in China, where all the botanicals in Table 1 are also included in the country’s *Pharmacopoeia*. Additionally, botanicals such as clove, ginkgo, and ginger are present in all four lists or databases, which suggests their widespread popularity and efficacy as both food and medicine.

## 3. Nutrients from These Botanical Food Ingredients with Kidney-Protective Effects

### 3.1. Flavonoids

Flavonoids are polyphenolic compounds that are widely distributed in a variety of botanical foods. The basic chemistry structure of flavonoids consists of two benzene rings with a phenolic hydroxyl group and a heterocyclic ring forming a C6-C3-C6 basic carbon framework [35]. The structure can be further categorized into flavanones, flavones, flavonols, flavanols, dihydroflavonols, and anthocyanins based on different functional groups and their positions [36]. It is one of the most bioactive and common compounds in food resources, especially botanicals. As demonstrated in Table 2, flavonoids that have been validated to have kidney-protective effects include quercetin [37,38,39], kaempferol [40], myricetin [41,42], isorhamnetin [43,44], fisetin [45,46], icariin [47,48], apigenin [49,50], baicalein [51,52], baicalin [53,54], nobiletin [55], vitexin [56,57], hesperidin [58,59], and hesperetin [60,61]. Quercetin and kaempferol appear in the majority of vegetables and fruits, which accounts for their status as the most prevalent dietary supplement ingredients. A review of the DSLD revealed that dietary supplements containing quercetin constituted 2.3% of the total products [29]. As illustrated in Table 2, citrus botanicals such as *Citrus medica* L. var. *sarcodactylis* Swingle and *Citrus reticulata* Blanco exhibit a notable content of flavonoids, including hesperidin, hesperetin, naringin, quercetin, and kaempferol [62,63,64]. The flavonoids present in citrus fruits have been demonstrated to possess potent antioxidant properties, making them a promising avenue of research for the development of novel therapeutic agents for the treatment of diabetes, neurodegenerative disorders, and kidney diseases such as AKI and diabetic nephropathy (DN) [65,66,67,68,69]. In addition to citrus, *Glycine max* (L.) Merr. (soybean) in Table 1 contains a unique kind of isoflavones called soy isoflavones, or “phytoestrogens” due to their structural similarity with 17-β-estradiol [70]. Though most of the studies of soy isoflavones fell into their endocrine regulation impacts, one recent study had discovered that the intake of soy isoflavones exhibited favorable effects on renal function and kidney morphology [71]. Puerarin, derived from *Pueraria lobata* (Willd.) Ohwi, is also an isoflavone. Recent studies have demonstrated its influence on the toll-like receptor (TLR) 4/MyD88 pathway and the M1 macrophage differential [72]. Later, flavonoids in *Pueraria thomsonii* Benth. were further found to decrease inflammation in the kidney by regulating the level of Clostridium in the gut [73]. Hesperidin from citrus botanicals could also alter gut microbiota against Zn-induced nephrotoxicity [58], indicating the potential of the gut–kidney axis.

### 3.2. Polysaccharides

Natural polysaccharides are a class of complex macromolecules that are formed from a variety of monosaccharide units and diverse glycosidic linkages. The monosaccharide units can be classified into several categories, including hexose, which includes glucose, galactose, and mannose; N-acetyl-hexose (HexNac), which encompasses GalNac and GluNac; pentose (e.g., xylose and arabinose); deoxyhexose (e.g., fucose); and numerous terminal modifications, such as phosphorylation, sulfation, and sialylation. Up to the present date, the identification and isolation still remain a critical challenge. However, the polysaccharides were found to be the principal compounds in some of the botanicals in Table 1 and the pharmacological effects of polysaccharides have also long been confirmed. Table 2 illustrates the representative polysaccharides from botanicals in Table 1 and Table S1, along with their impact on kidney diseases.

*Laminaria japonica* Aresch. is a widespread food ingredient in China with a significant amount of polysaccharides. It contains polysaccharides weighing from 5 kDa to 10^4^ kDa [231] with great potential in anti-fibrosis effects, which revealed its capacity to treat CKD [106]. In many kidney diseases including the DN model [104,105], DIN model [103,106], and IRI model [102], polysaccharides from *Laminaria japonica* Aresch. showed strong impact on fibrosis- and apoptosis-related molecules like TGF-β, α-SMA, and Bax/Bcl-2. Polysaccharides are also identified to be the primary compounds in *Polygonatum sibiricum Red* [232]. Polygonat Rhizomai polysaccharides were discovered to be protective against uranium-induced AKI with 141 kDa polysaccharides in them [17]. The polysaccharides from other traditional kidney-protective botanical food like *Lycium barbarum* L. [101] and *Astragalus membranaceus* [19,96,97] showed protective effects against DIN as well.

In consideration of the gut–kidney axis, polysaccharides derived from these botanical ingredients also play a significant role in the intricate interplay of gut–kidney crosstalk. The polysaccharides derived from *Phyllostachys nigra* [20], *Paeonia × suffruticosa* (Moutan Cortex) [111], and *Astragalus membranaceus* (Fisch.) Bge. [97] have been observed to possess the capacity to regulate the gut–kidney axis, thereby conferring benefit to the kidney. The mechanisms involved in remodeling the gut microbiota composition include an increase in the abundance of probiotics, such as *Lactobacillales*, and an upregulation of SCFAs.

### 3.3. Terpenoids

Terpenoids, which are derived from isoprene, encompass a diverse range of chemical compounds, including monoterpenoids, sesquiterpenoids, diterpenoids, sesterterpenes, triterpenoids, and polyterpenoids [233]. Considering the ingredients in Table 1 and Table S1, *Panax ginseng* C. A. Mey. (ginseng), *Glycyrrhiza uralensis* Fisch. (Liquorice), *Alisma orientatle* (Sam.) Juzep., and *Ganoderma lucidum* (Leyss. ex Fr.) Karst. (Ganoderma) are representative for their great amount and variety of terpenoids.

Ganoderma triterpenoids isolated from Ganoderma are some of the major chemical constituents in Ganoderma and could be further divided into C30 (ganoderic acid, ganodermanontriol, applanoxidic acids, and their derivatives), C27 (lucidenic acid, ganolactone, and their derivatives), and C24 (lucidones and their derivatives), and others according to their skeleton carbons [234]. Among the compounds identified, ganoderic acids had been proven to possess protective properties in the context of ADPKD [159], renal fibrosis [160], and IRI [161]. Licorice is another ingredient that has been demonstrated to possess renal-protective terpenoids, the most prominent of which are glycyrrhizic acid and glycyrrhetinic acid [235]. Glycyrrhizic acid possesses surprising protective effects in DN [162,163] and DIN including tacrolimus [164], contrasts [165], and triptolide [166]-induced kidney injury. Similar effects were also found in glycyrrhetinic acid in DN [162] and IRI [167]. *Alisma orientatle* (Sam.) Juzep. and *Panax ginseng* C. A. Mey. are two botanicals in Table S1, which means that they are not considered daily food but could be added to dietary supplements. However, they have been traditionally used to treat renal disorders in TCM, especially *Alisma orientatle* (Sam.) Juzep. Alisols and their derivatives are tetracyclic triterpene alcohols isolated from *Alisma orientatle* (Sam.) Juzep. and play a significant role in its pharmacological effects [236]. Recent studies had demonstrated the potential of alisols in protecting the kidney from DPP-induced injury and IRI by targeting the farnesoid X receptor and soluble epoxide hydrolase [148,149]. Additionally, alisol B 23-acetate was observed to regulate the gut microbiota, thereby improving renal function in CKD mice [150]. Ginsenosides, the representative compounds in ginseng, are a big family of triterpenoid saponins with a four-ring rigid steroid skeleton [237]. Early in 1998, a study revealed that ginsenoside Rd could protect against IRI by affecting proximal tubule cells [238]. Later, numerous studies had demonstrated that ginsenosides could alleviate AKI [151,152], renal carcinoma [153,154], DN [155,156], and renal fibrosis [157,158].

Iridoids constitute a large and distinctive class of monoterpenoids that are ubiquitous in botanical sources and have been demonstrated to exhibit notable bioactivity [239]. Iridoids are receiving increasing attention for their great pharmacological effects in the liver, kidney, and nervous disorders [240,241,242,243]. Catalpol, one of the most common iridoids, for example, showed strong antioxidation and anti-inflammation effects in DN-, CKD-, and DIN-induced AKI models [115,116,117,118,119,120,121]. *Cornus officinalis* Sieb. et Zucc. was found to be abundant in iridoids [244], in which loganin and morroniside were shown to be protective for the kidney [122,123,124,125,133]. The representative terpenoids including triterpenoids and iridoids from botanicals with medicine–food homology are also shown in Table 2.

### 3.4. Alkaloids

Isolated from natural herbs, alkaloids are a diverse group of naturally occurring organic compounds that primarily contain basic nitrogen atoms [245]. Structurally, they could be characterized by a heterocyclic ring that incorporates nitrogen, and they often possess complex and varied molecular frameworks, for example, isoquinoline, pyrrolidines, pyridines, indole, and others. Dietary botanicals also contain variable bioactive alkaloids for renal protection. Table 2 provides a list of the principal representative alkaloids with kidney-protective effects derived from the botanicals included in Table 1 and Table S1.

Tetramethylpyrazine, the characteristic alkaloid of *Ligusticum sinense* Chuanxiong, has been clinically utilized to inhibit platelet aggregation and reduce blood viscosity [246]. Its renal-protective effects were also validated in multiple models including DIN-induced AKI [168,169], IRI [170], and DN [171,172] with classical mechanisms targeting inflammation and oxidation markers. Leonurine from *Leonurus japonicus* Houtt. also showed protective effects in lipopolysaccharide-, DDP-, and vancomycin-induced AKI [173,174,175,176,177]. Besides AKI, trigonelline, the characteristic alkaloid in *Trigonella foenum-graecum* L. (Fenugreek) and *Coffea arabica* (coffee) was demonstrated to maintain a strong protective effect on oxalate nephropathy (ON) [191,192], which is closely related to diet oxalate. *Nelumbo nucifera* Gaertn. is well known for its abundant alkaloids, especially in its seeds. Liensinine, isoliensinine, neferine, and nuciferine are all alkaloids with kidney-protective effects isolated from *Nelumbo nucifera* Gaertn. Besides AKI and DN, neferine was also revealed to alleviate hyperuricemic nephropathy by targeting the inflammasome pathway [208]. Another alkaloid that has been widely studied for its protective effects on the kidneys is berberine. This isoquinoline alkaloid was initially isolated from *Hydrastis canadensis* and is primarily found in *Coptis chinensis*. Although these botanicals are not typically included in dietary regimens, berberine itself is a frequently utilized compound in dietary supplements. In DSLD, dietary supplements containing berberine reached 865 records. The renal-protective effects and mechanism of berberine have long been studied since 2011 in the DN model [247,248]. Later, the extraordinary effects of berberine were found in multiple nephropathy models including DIN-induced AKI [178,179,180,181,182], IRI-induced AKI [183,184,185], DN [186,187,188], and UUO-induced renal fibrosis [189]. The mechanisms involved in the treatment of berberine contained traditional anti-inflammation, oxidation, apoptosis, and also the regulation of gut bacteria. With the increasing interest in the gut microbiota, the interaction between the gut microbiota and berberine in the treatment of renal diseases has shown great promise. Recent studies demonstrated that berberine could improve chronic kidney disease by inhibiting the production of enterogenous toxins such as trimethylamine oxide (TMAO) and p-cresol (pCS), which are metabolites from gut microbiota [190]. Berberine could also increase the abundance of *Coprococcus*, *Bacteroides*, *Akkermansia*, and *Prevotella* to alleviate UAN [249,250,251]. A growing body of evidence suggests that the mechanisms of renoprotective effects through the gut–renal axis deserve further investigation.

Though many alkaloids possess renal-protective effects, a few studies have yielded contradictory results for the same compounds, for example, matrine from *Sophora japonica* L. Some studies [252,253] found that matrine could lead to kidney injury with mitochondria dysfunction and oxidation while another study demonstrated its protective effect on the DDP-induced AKI model [198]. Its derivate, oxymatrine, was also found to alleviate gentamicin-induced AKI [199], IRI [200], and renal fibrosis [254]. It again reinforces the necessity for the scientific research and regulation of the dietary utilization of botanicals with medicine–food homology.

### 3.5. Others

In addition to the aforementioned classes of compounds, botanicals with medicine–food homology also contain many other abundant bioactive nutrients including various kinds of polyphenols and anthraquinones, among which some showed great potential in benefiting the kidney as shown in Table 1. Simple phenylpropanoids are a kind of natural polyphenols with a three-carbon side chain linked to a phenyl ring, forming a C6-C3 skeleton. The primary structure also makes them precursors to many other natural compounds, such as flavonoids, lignans, and polyphenols [255]. Simple phenylpropanoids, including the widely distributed ferulic acid and chlorogenic acid, were both found to remodel the composition of the gut microbiota, thereby alleviating UAN [227,228,229]. Curcumin, the most bioactive and abundant polyphenol [256] in *Curcuma longa* L., has been extensively investigated for its potent antioxidant effects [213]. Together with its derivatives, curcumin displayed potential in treating LN [212], DIN-induced AKI [211], DN [215], and even focal segmental glomerulosclerosis (FSGS) [214]. Additionally, research has indicated that curcumin may mitigate renal impairment by modulating the microbiota in conditions such as CKD and UAN [257,258]. Sesamin, a lignan from dietary sesame, was also demonstrated to decrease uremic toxins by inhibiting the gut microbiota indole pathway [223], indicating importance of the gut–kidney axis. Anthraquinone compounds, for example, emodin and its derivatives, are the most important chemical compounds in *Rheum palmatum* L., which has long been used in TCM and added to dietary supplements [259]. The anthraquinone compounds in *Rheum palmatum* L. also possess renal-protective effects on DN, CKD, and UUO models, mainly targeting TGF-β secretion [216,217,218,219,220]. Interestingly, emodin was found to decrease uremic toxins as well by targeting gut microbiota [260]. An increasing body of evidence is emerging that demonstrates the significance of the gut–kidney axis in relation to dietary nutrients.

## 4. Mechanisms Involved in the Kidney-Protective Effects of Botanical Ingredients with Medicine–Food Homology

### 4.1. Antioxidation, Anti-Inflammation, and Anti-Fibrosis

Previous studies had focused on the inflammation/oxidation-oriented mechanisms, which are key pathological changes in kidney diseases. Oxidative stress is characterized by an initial injury in the kidney due to the activities of intra- and extracellular oxygen-derived radicals and the resultant inflammatory response [261]. When the kidney is exposed to harmful stimuli, ROS such as superoxides and hydroxyl radicals are produced in excess and interact with the molecular components and functions of a nephron such as the cell membrane, DNA methylation, histone modifications, and micro-RNAs, which are crucial mechanisms for fetal programming [262]. The production and scavenging of oxygen-derived radicals are in a dynamic balance through enzymatic (SOD, CAT, HO-1) and non-enzymatic (vitamin C, vitamin E, glutathione) antioxidant systems [263]. To deal with increased oxidative stress, several redox signaling response cascades, including Nrf2, could be activated to regulate this antioxidative gene expression [263]. Due to the existence of the oxygen shunt diffusion, the kidney is highly susceptible to oxidative stress and hypoxia [264]. Abnormalities in oxidative stress have been observed in a range of kidney diseases, including CKD, DN, and DIN-induced AKI and CKD [265,266,267,268].

The critical role of inflammation in the development of AKI, CKD, and DN was also widely confirmed in animal and clinical studies. The inflammation in the kidney is activated by various mechanisms including endothelial injury, drugs, and infection and causes glomerular, interstitial, and vascular damage [269]. The process of neutrophil infiltration is accompanied by an increase in multiple pro-inflammatory chemotaxes, adhesion factors, and chemokines, including ICAM-1, P- and E-selectin, and IL-1β, IL-6, IL-18, and TNF-α. This process is strongly correlated with fibrosis, autophagy, oxidative stress, and mitochondrial dysfunction [270]. In primary glomerular disease including acute glomerulonephritis, IgA nephropathy, and nephrotic syndrome, complement activation and immune complex deposition, which elicit strong inflammation, were also identified as key pathogenesis [271,272]. Pro-inflammatory signaling pathways include the TLR4/MyD88 signaling pathway, which is also closely related to gut microbiota due to its recognition of pathogen-associated molecular patterns [273]. The activation of TLR4 could lead to the upregulation of another key pro-inflammatory signal, the NF-κB-related pathway [273], which is also the target for most of the anti-inflammation effects in Table 2.

Fibrosis represents another pivotal pathological process following inflammation, especially chronic inflammation. With continuous inflammatory stimulation, various stromal cells in the kidney are transformed to myofibroblasts, which contribute to excessive extracellular matrix production and deposition in the renal parenchyma, eventually leading to loss of renal function [274]. The increase in the secretion of TGF-β and the fibroblast growth factor (EGF) and the secretion of type I collagen, fibronectin, chondroitin sulfate, and other components of the extracellular matrix (ECM) are also directly involved in the epithelial–mesenchymal transition (EMT) and fibrosis [275]. Recent studies had discovered that MMP also contributes to renal fibrosis [276]. There are several severe molecules and pathways involved in the complicated process of renal fibrosis, for instance, the Wnt/β-catenin and TGF-β1/Smad pathways, which are also key targets for botanicals. As the most vital target, Smad3-mediated TGF-β stimulation not only induces collagen production and the inhibition of ECM degradation, but also depresses fatty acid oxidation, leading to a profibrotic phenotype [275].

Botanicals with medicine–food homology happen to be abandoned in natural antioxidant, anti-inflammation, and anti-fibrosis compounds regulating the above pathways and inflammatory cytokines as shown in Table 2. The majority of the mechanisms underlying the protective effects are associated with these mechanisms, underscoring the significant potential and importance of incorporating daily dietary botanicals and supplements into one’s routine.

### 4.2. Regulating the “Gut–Kidney Axis”

In addition to the classical molecular pathological processes centered on inflammation, oxidative stress, and cellular autophagy and apoptosis, a large number of studies have recently revealed a relationship between intestinal disorders and renal disease, a link that has been increasingly emphasized and investigated under the term “gut–kidney axis” [4]. It has been suggested that the gut–renal axis can be subdivided into metabolism-dependent and immunologic pathways [277]. Metabolism-dependent pathways are mainly mediated by metabolites produced by the gut microbiota that have the ability to modulate host physiological functions. For example, alterations in the gut microbiota can lead to intestinal metabolism and dysfunction, thereby increasing uremic toxin production and renal dysfunction [190,278]. On the other hand, kidney disease also affects the composition of gut bacteria and could cause gastrointestinal dysfunction [279,280]. In addition to uremic toxins like pCS, the gut microbiota produces a large number of physiologically active substances such as SCFAs, and bile acids (BAs), which play an important role in the regulation of renal disease and function [4]. Studies have found a reduction in SCFA-producing gut bacteria including *Lactobacillaceae* and *Prevotella* species in ESRD patients compared to healthy individuals [281,282]. However, in some models of kidney disease, a high-fiber diet that promotes the growth of SCFA-producing bacteria and direct supplementation with SCFAs attenuated renal fibrosis [283]. As for the immune pathway, components of the immune system (e.g., lymphocytes, monocytes, and cytokines) play a key role in the communication between the gut and the kidney [284]. A growing number of studies have demonstrated that gut flora play a crucial role in mucosal immunity and systemic inflammation, and that changes in gut flora induce changes in inflammatory cytokine profiles in the bone marrow [285], which are further associated with the development of autoimmune nephropathy [286]. Lymphocytes could even migrate from the gut to the injured kidney via the chemokine pathway [284]. Recent studies have also found that the aberrant galactose-deficient IgA, which is key in the pathogenesis of IgA nephropathy, is associated with aberrant hydrolysis by intestinal bacteria, and is recognized in vivo through the metabolism of the intestinal bacteria exposing aberrant antigenic epitopes, which leads to the formation of immune complexes and renal deposition [286].

As attention is increasingly focused on the gut microbiota, their interaction with botanicals has been discovered and showed a promising future [287,288,289,290,291,292]. As shown in Table 2, almost all kinds of compounds have the potential in regulating gut microbiota. Panax notoginseng saponins, the bioactive components of a well-known and widely used botanical, *Panax notoginseng*, in Table S1, could regulate intestinal microorganisms and therefore ameliorate inflammation and fibrosis. Similarly to terpenoids, the renal protection by oleanic acid was also found to be associated with its regulation on gut microbiota [147]. Berberine had also been found to ameliorate chronic kidney disease through inhibiting the production of gut-derived uremic toxins, for example, TMAO and pCS in the gut microbiota [190]. *Astragalus membranaceus*, of which the anti-inflammation and fibrosis effects were confirmed, was also found to influence the gut microbiota, with *Akkermansia muciniphila* and *Lactobacillus* being the main driving bacteria [97]. More and more renal-protective effects of botanicals are found to be associated with the gut–kidney axis. Meanwhile, the gut microbiota exhibit rapid alterations in response to daily diets [293,294], indicating the potential for dietary nutrients to intervene in the gut–kidney axis.

## 5. Conclusions and Perspective

**Figure 2:**
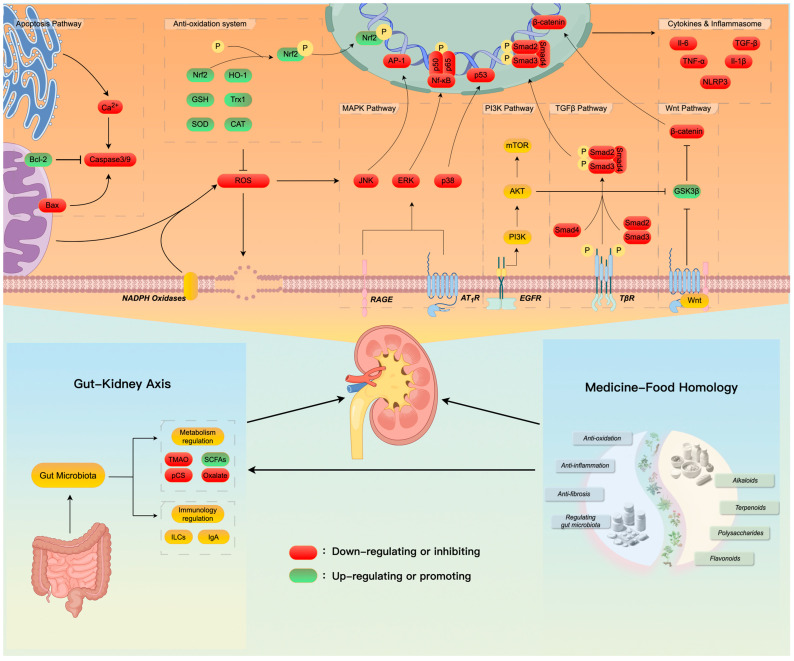
Mechanisms involved in the interplay of dietary medical botanicals and kidney function, drawn by authors using Figdraw (www.figdraw.com).

The concept of “food as medicine” underpins the traditional use of these botanicals, especially within the framework of TCM. This approach, which integrates dietary and medicinal uses, provides a unique and holistic perspective on disease prevention and health maintenance. The review emphasizes that many botanicals have been traditionally used for both food and therapeutic purposes, demonstrating their dual benefits. The diverse bioactive compounds found in these botanicals, such as flavonoids, polysaccharides, saponins, alkaloids, and polyphenols, demonstrate a range of protective effects on renal health. These effects are primarily mediated through antioxidation, anti-inflammation, anti-fibrosis, the modulation of the immune response, and enhancement in mitochondrial function as shown in Figure 2. The integration of these botanicals into modern therapeutic regimes could offer a complementary approach to conventional treatments for kidney diseases, which often focus on symptom management rather than disease reversal. The promising results from experimental studies suggest that incorporating these natural compounds into dietary habits or as supplements could contribute to better renal health outcomes.

A particularly notable aspect highlighted in this review is the interplay between these botanicals and the gut–kidney axis. This emerging area of research emphasizes the complex relationship between gut microbiota and renal function. Botanical compounds can influence the composition and activity of gut microbiota, which in turn affects systemic inflammation, immune responses, and metabolic processes relevant to kidney health. For instance, the modulation of gut microbiota by polysaccharides and polyphenols can lead to the production of beneficial metabolites, such as short-chain fatty acids, which have protective effects on the kidneys. Considering the close relation between daily diets and gut microbiota, the potential for dietary nutrients to intervene in the gut–kidney axis is worth deeper exploration.

In summary, botanical ingredients with medicine–food homology present a valuable resource for developing new strategies in the prevention and management of kidney diseases. By leveraging their effects on the gut–kidney axis and other pathways, these botanicals hold promise for enhancing renal health and improving patient outcomes. Continued research and clinical validation are essential to fully realize their potential in this domain.

## Figures and Tables

**Table 1:** The full list of botanicals in the China index for medicine–food homology.

Latin Name	English Common Name	EMA Herbal Medicine	EFSA Compendium	NIH DSLD
*Eugenia caryophyllata* Thunb.	Clove	√	√	√
*Illicium verum* Hook. f.	Star anise		√	√
*Canavalia gladiata* (Jacq.) DC.	Sword bean			√
*Foeniculum vulgare* Mill.	Fennel	√	√	√
*Cirsium setosum* (Willd.) MB.	Thistle			
*Dioscorea opposita* Thunb.	Chinese yam			√
*Crataegus pinnatifida* Bge. var. *major* N.E.Br./*Crataegus pinnatifida* Bge.	Hawthorn	√	√	√
*Portulaca oleracea* L.	Purslane		√	√
*Prunus mume* (Sieb.) Sieb. et Zucc.	Japanese apricot		√	√
*Chaenomeles speciosa* (Sweet) Nakai	Flowering quince		√	
*Cannabis sativa* L.	Hemp	√		√
*Citrus aurantium* L. var. *amara* Engl.	Bitter orange			
*Polygonatum odoratum* (Mill.) Druce	Solomon’s seal		√	
*Glycyrrhiza uralensis* Fisch./*Glycyrrhiza inflata* Bat./*Glycyrrhiza glabra* L.	Licorice	√	√	√
*Angelica dahurica* (Fisch. ex Hoffm.) Benth. et Hook. f./*Angelica dahurica* (Fisch. ex Hoffm.) Benth. et Hook. f. var. *formosana* (Boiss.) Shan et Yuan	Chinese angelica		√	
*Ginkgo biloba* L.	Ginkgo	√	√	√
*Dolichos lablab* L.	Hyacinth bean		√	√
*Dimocarpus longan* Lour.	Longan		√	√
*Cassia obtusifolia* L./*Cassia tora* L.	Sicklepod		√	
*Lilium lancifolium* Thunb./*Lilium pumilum* DC.	Tiger lily		√	√
*Myristica fragrans* Houtt.	Nutmeg		√	√
*Cinnamomum cassia* Presl	Cassia	√	√	√
*Phyllanthus emblica* L.	Amla		√	
*Citrus medica* L. var. *sarcodactylis* Swingle	Fingered citron			
*Prunus armeniaca* L. var. *ansu* Maxim/*Prunus sibirica* L./*Prunus mandshurica* (Maxim) Koehne/*Prunus armeniaca* L.	Chinese apricot		√	√
*Hippophae rhamnoides* L.	Sea buckthorn			
*Euryale ferox* Salisb.	Euryale ferox		√	
*Zanthoxylum schinifolium* Sieb. et Zucc./*Zanthoxylum bungeanum* Maxim.	Sichuan pepper			√
*Vigna umbeuata* Ohwi et Ohashi/*Vigna angularis* Ohwi et Ohashi	Azuki bean			
*Hordeum vulgare* L.	Barley		√	√
*Laminaria japonica* Aresch./*Ecklonia kurome* Okam.	Kunbu			
*Ziziphus jujuba* Mill.	Jujube		√	√
*Siraitia grosvenorii* (Swingle.) C.Jeffrey ex A. M. Lu et Z. Y.Zhang	Luohanguo siraitia fruit		√	
*Prunus japonica* Thunb.	Pruni semen		√	
*Lonicera japonica* Thunb.	Japanese honeysuckle		√	
*Canarium album* Raeusch.	White canarium		√	
*Houttuynia cordata* Thunb.	Fish mint		√	
*Zingiber officinale* Rosc.	Ginger	√	√	√
*Hovenia dulcis* Thunnb./*Hovenia acerba* Lindl*./Hovenia trichocarpa* Chun et Tsiang	Japanese raisin tree		√	
*Lycium barbarum* L.	Goji berry		√	√
*Gardenia jasminoides* Ellis	Cape jasmine		√	√
*Amomum villosum* Lour./*Amomum villosum* Lour. var. *xanthioides* T. L. Wu et Senjen/*Amomum longiligulare* T. L. Wu	Villus amomum		√	√
*Sterculia lychnophora* Hance	Malva nut tree		√	√
*Poria cocos* (Schw.) Wolf	Poria, fu ling			
*Citrus medica* L./*Citrus wilsonii* Tanaka	Citron		√	√
*Mosla chinensis* Maxim./*Mosla chinensis* Maxim. cv. Jiangxiangru	Chinese mosla		√	
*Prunus persica* (L.) Batsch/*Prunus davidiana* (Carr.) Franch.	Peach		√	√
*Morus alba* L.	White mulberry		√	√
*Citrus reticulata* Blanco	Mandarin orange		√	√
*Platycodon grandiflorum* (Jacq.) A. DC.	Balloon flower		√	√
*Alpinia oxyphylla* Miq.	Sharp-leaf galangal		√	√
*Nelumbo nucifera* Gaertn.	Lotus leaf		√	√
*Raphanus sativus* L.	Radish seed		√	√
*Alpinia officinarum* Hance	Lesser galangal		√	√
*Lophatherum gracile* Brongn.	Lophatherum		√	√
*Glycine max* (L.) Merr.	Soybean	√	√	√
*Chrysanthemum morifolium* Ramat.	Chrysanthemum		√	√
*Cichorium glandulosum* Boiss. et Huet/*Cichorium intybus* L.	Chicory		√	
*Polygonatum kingianum* Coll.et Hemsl./*Polygonatum sibiricum Red./Polygonatum cyrtonema* Hua	Polygonati rhizoma		√	
Sinapis alba L.	White mustard		√	√
*Perilla frutescens* (L.) Britton	Perilla		√	√
*Pueraria lobata* (Willd.) Ohwi/*Pueraria thomsonii* Benth.	Kudzu		√	√
*Sesamum indicum* L.	Sesame		√	√
*Piper nigrum* L.	Black pepper		√	√
*Sophora japonica* L.	Sophora		√	√
*Taraxacum mongolicum* Hand. Mazz./*Taraxacum borealisinense* Kitam.	Dandelion	√	√	√
*Torreya grandis* Fort.	Torreya		√	√
*Ziziphus jujuba* Mill. var. *spinosa* (Bunge) Hu ex H. F. Chou	Chinese date			
*Imperata cylindrical* Beauv. var. *major* (nees) C. E. Hubb.	Cogongrass			
*Phragmites communis* Trin.	Reed		√	√
*Mentha haplocalyx* Briq.	Chinese mint	√	√	√
*Coix lacryma-jobi* L. var. *mayuen* (Roman.) Stapf	Job’s tears		√	√
*Allium macrostemon* Bge./*Allium chinense* G. Don	Chinese onion		√	
*Rubus chingii* Hu	Chinese raspberry	√	√	√
*Pogostemon cablin* (Blanco) Benth./*Agastache rugosus* (Fisch. et Mey.) O. Ktze.	Patchouli		√	√
*Angelica sinensis* (Oliv.) Diels	Angelica root	√	√	√
*Kaempferia galanga* L.	Aromatic ginger		√	√
*Crocus sativus* L.	Saffron		√	√
*Amomum tsao-ko* Crevost & Lemarié	Cardamom		√	√
*Curcuma longa* L.	Turmeric	√	√	√
*Piper longum* L.	Long pepper		√	√
*Codonopsis pilosula* (Franch.) Nannf./*Codonopsis pilosula* Nannf. var. *modesta* (Nannf.) L. T. Shen/*Codonopsis tangshen* Oliv.	Dangshen		√	√
*Cistanche deserticola* Y. C. Ma	Desert cistanche		√	√
*Dendrobium officinale* Kimura et Migo	Chinese orchid		√	√
*Panax quinquefolius* L.	American ginseng		√	√
*Astragalus membranaceus* (Fisch.) Bge.	Mongolian milkvetch (huangqi)		√	√
*Ganoderma lucidum* (Leyss. ex Fr.) Karst./*Ganoderma sinense* Zhao, Xu et Zhang	Ganoderma			
*Cornus officinalis* Sieb. et Zucc.	Japanese cornel dogwood		√	
*Gastrodia elata* Bl.	Gastrodia		√	√
*Eucommia ulmoides* Oliv.	Hardy rubber tree		√	√

EMA: European Medicines Agency; EFSA: European Food Safety Authority; NIH: National Institutes of Health; DSLD: Dietary Supplement Label Database; √: Indicating this botanical also appears in the index or database of ingredients; var.: Variety; f.: Form; cv.: Cultivar.

**Table 2:** Nutrients from these botanical food ingredients with kidney-protective effects.

Botanical Resources	Compounds	Models	Effects	Ref.
**Flavonoids**				
*Allium macrostemon* Bge., *Crocus sativus* L., *Plantago asiatica* L. ^S^	Quercetin	DN rats, ochratoxin A-induced AKI mice	↓ PI3K/AKT signaling ↑ Nrf2/HO-1, fatty acid oxidation	[37,38,39,74,75]
*Glycine max* (L.) Merr., *Lonicera japonica* Thunb., *Ginkgo biloba* L., *Lycium barbarum* L., *Plantago asiatica* L. ^S^	Kaempferol	CLP-induced AKI mice, DN mice, DOX-induced AKI mice	↓ ICAM-1, VCAM-1, MCP-1, caspase-3, Bax, MAPK signaling, TGF-β1, and α-SMA ↑ Bcl-2, SOD, and GSH	[40,76,77,78]
*Myricaceae*, *Polygonaceae*, *Primulaceae*, *Pinaceae*, *and Anacardiaceae*	Myricetin	DN mice, EG-induced AKI mice	↓ IL-1β, TNF-α, ROCK1/ERK/P38 signaling, and NF-κB signaling ↑ Nrf2, CAT, and SOD	[41,42,79]
*Ginkgo biloba* L., *Hippophae rhamnoides* L., *Citrus reticulata* Blanco, *Citrus aurantium* L., *and Citrus medica* L.	Isorhamnetin	LPS-induced AKI mice	↓ IL-1β, IL-6, and TNF-α and M1 macrophage ↑ M2 macrophage	[43,44]
*Epimedium brevicornu* Maxim. ^S^	Icariin	Adenine/UUO-induced CKD rats, DOX-induced AKI mice	↓ TGF-β, α-SMA, and E-cadherin ↑ Nrf2/HO-1, SOD, CAT	[47,48,80,81,82]
*Plantago asiatica* L. ^S^	Apigenin	DOX/EG/oxonate-induced AKI mice, UAN mice	↓ Oxidative and nitrosative stress, IL-6, TNF-α, NLRP3, caspase-1, IL-1β, TGF-β, Wnt/β-catenin signaling	[49,50,83,84,85,86,87]
*Citrus reticulata* Blanco, *Citrus aurantium* L., *Citrus medica* L.	Nobiletin	IRI-induced AKI mice	↑ PI3K/AKT signaling	[55]
*Crataegus pinnatifida* Bge.	Vitexin	UUO-induced CKD mice, oxalate-induced AKI mice	↓ NLRP3, caspase-1, and IL-1β ↑ Nrf2/HO-1, SOD, GSH	[56,57]
*Citrus reticulata* Blanco, *Citrus aurantium* L., *Citrus medica* L.	Hesperidin, hesperetin	LPS/Zn-induced AKI mice, DDP-induced HK-2 cells	↓ p53, caspase-3 ↑ Nrf2/HO-1, SOD, GSH, and CAT Regulating gut microbiota	[58,59,60,61]
*Plantago asiatica* L. ^S^	Luteolin	Cd/HgCl_2_/K_2_Cr_2_O_7_-induced AKI mice, LN mice	↓ HIF-1α, α-SMA, collagen I, and fibronectin ↑ Nrf2/HO-1, GSH, SOD, CAT, and AMPK/mTOR autophagy	[88,89,90,91,92]
*Pueraria lobata* (Willd.) Ohwi	Puerarin	DN mice, UUO-induced CKD mice	↑ Nrf2, cAMP/PKA/CREB ↓ TLR4/MyD8, and M1 macrophage	[72,93,94,95]
**Polysaccharides**				
*Astragalus membranaceus* (Fisch.) Bge.	/	DDP-induced AKI mice	↓ ROS generation and mitochondrial vacuolation	[19]
	/	LPS-induced AKI mice	↓ Caspase-3/9 and Bax ↑ Bcl-2	[96]
	/	LPS-induced AKI mice	Regulating gut microbiota ↑ SCFAs	[97]
*Bletilla striata* ^S^	Mw: 260 kDa	Ang II-induced HMCs	↓ ROS generation and NOX4	[18]
	/	TGF-β-induced HMCs	↓ TGF-β, and α-SMA	[98]
*Ganoderma lucidum* (Leyss. ex Fr.) Karst.	Mw: 72.9 kDa; Ara:Gal:Rha:Glc = 0.08:0.21:0.24:0.47	DN mice	↓ Collagen-1, fibronectin, α-SMA, TGF- β, and MAPK/NF-κB signaling	[99]
	/	IRI-induced AKI mice	↓ p53, caspase-3, Bax, cytochrome c, and ER stress ↑ Bcl-2	[100]
*Lycium barbarum* L.	Ara:Gal:Glc:GalA:Man:Rha = 12.25: 8.66: 7.66: 2.86: 1.70: 1.00	DN mice	↓ TNF-α, IL-1β, IL-6, and NF-κB signaling	[16]
	/	Lead-induced AKI mice	↓ Bax and caspase-3 ↑ Bcl-2	[101]
*Laminaria japonica* Aresch./*Ecklonia kurome* Okam.	Mw: 7 kDa	IRI-induced AKI mice	↓ p53, Bax, MMP, and cytochrome c ↑ Bcl-2	[102]
	Mw: 1960 kDa	DOX-induced AKI mice	↓ TNF-α, IL-1β, MCP-1, and podocyte injury	[103]
	Mw: 8.84 kDa; Fuc:Gal:Man:Glc:Rha:Xyl = 1:0.057:0.041:0.008:0.029:0.019	DN mice	↓ collagen-1, fibronectin, and α-SMA	[104]
	Mw: 7 kDa	DN mice	↓ α-SMA, fibronectin, and TGF-β/Smad ↑ E-cadherin	[105]
	/	DOX-induced CKD mice	↓ α-SMA, fibronectin, and TGF-β/Smad	[106]
	Mw: 7.774 kDa	AGE-induced HRMCs	↓ Fibronectin	[107]
	Mw: 8.84 kDa	TGF-β1- or FGF-2-induced HK-2 cells	↓ α-SMA, MMP9, and EMT	[108]
*Polygonatum kingianum* Coll.et Hemsl./*Polygonatum sibiricum* Red./*Polygonatum cyrtonema* Hua	Mw: 141 kDa; Gal:GalA:Ara:Glc = 57.67:26.82:4.59:4.54	Uranium-induced HK-2 cells	↓ ROS ↑ GSK-3β/Fyn/Nrf2	[17]
*Panax ginseng* C. A. Mey. ^S^	Glc:Gal:Ara:GalA:Rha:Man = 76.7:6.5:5.1:9.2:1.4:1.1	DDP-induced AKI mice	↓ p53, caspase-3, caspase-6, and ER stress by PERK/eIF2α/ATF4 signaling	[109]
*Paeonia × suffruticosa* ^S^	Mw: 164 kDa; D-Glc:L-Ara = 3.31:2.25;	DN rats	↓ TGF-β, ICAM-1, and VCAM-1	[110]
	Mw: 164 kDa; Ara = 3.31:2.25	DN rats	Regulating gut microbiota ↑ SCFAs	[111]
*Plantago asiatica* ^S^	/	Adenine-induced rats, UAN	↓ IL-6, TNF-α, NLRP3, and caspase-1	[112]
*Dendrobium officinale* Kimura et Migo	/	HG-induced HK-2 cells, db/db mice	↓ TGF-β, and α-SMA ↑ SIRT1	[113]
*Salvia miltiorrhiza* Bunge ^S^	/	Florfenicol-induced AKI broilers	↓ p53, caspase-3 ↑ Nrf2/HO-1	[114]
*Phyllostachys nigra* ^S^	Mw: 34 kDa	DN mice	Regulating gut microbiota ↑ *Lactobacillales*	[20]
**Terpenoids**				
*Rehmannia glutinosa*^S^, *Plantago asiatica^S^*, *Scrophularia ningpoensis*^S^, *and Crocus sativus* L.	Catalpol	DN mice, DOX/Ang II/Fru/DDP-induced AKI mice, adenine-induced CKD mice	↓ TRPC6, NF-κB, TGF-β1/Smad, TLR4/MyD88 signaling, RAGE/RhoA/ROCK signaling ↑ AMPK signaling	[115,116,117,118,119,120,121]
*Cornus officinalis* Sieb. et Zucc.	Loganin	DN mice, IRI/DOX/CLP-induced AKI mice	↓ NLRP3, AGE/RAGE signaling ↑ Nrf2/HO-1	[122,123,124,125]
*Gardenia jasminoides* Ellis, *Eucommia ulmoides* Oliv., *Rehmannia glutinosa* ^S^, *and Scrophularia ningpoensis* ^S^	Geniposide	DN mice, CLP-induced AKI mice, H_2_O_2_-induced HK-2 cells	↓ ICAM-1, TNF-α, IL-1, IL-6, NF-κB, and NETs ↑ GSK3β, AMPK-PI3K/AKT, Bcl-2 Regulating gut microbiota	[126,127,128,129,130,131,132]
*Cornus officinalis* Sieb. et Zucc.	Morroniside	H_2_O_2_-induced podocytes	↓ NOX4	[133]
*Canarium album* Raeusch.	Oleuropein	DN mice, acrylamide-induced AKI mice	↓ TNF-α, IFN-γ, IL-2, IL-6, and IL-17α ↑ SOD, GSH-Px, and CAT	[134,135]
*Poria cocos* (Schw.) Wolf.	Poricoic acid (A, B, C, D, E, F, G, H, AM, AE, BM, DM, and derivatives)	UUO-induced CKD mice	↓ MMP-13, Wnt/β-catenin, TGF-β/Smad3/MAPK ↑ AMPK, Nrf2	[136,137,138,139,140,141,142]
*Ligustrum lucidum* Ait. ^S^	Oleanic acid	UUO-induced CKD mice, DN mice	↓ NF-κB/TNF-α, TGF-β ↑ Nrf2/SIRT1/HO-1 Regulating gut microbiota	[143,144,145,146,147]
*Alisma orientatle* (Sam.) Juzep. ^S^	Alisol (A, B, and derivatives)	IRI/DDP-induced AKI mice, UUO-induced CKD mice	↓ ICAM-1, MCP-1, COX-2, iNOS, IL-6, TNF-α, FXR activation, TGF-β/Smad3 ↑ SOD and GSH and HO-1	[148,149,150]
*Panax ginseng* C. A. Mey.	Ginsenosides	DDP-induced AKI mice, renal carcinoma, DN mice, UUO-induced CKD mice	↓ ER stress, lipid peroxidation, PPARγ, and NOX4-MAPK pathways and TGF-β	[151,152,153,154,155,156,157,158]
*Ganoderma lucidum* (Leyss. ex Fr.) Karst.	Ganoderic acid, ganodermanontriol, lucidenic acid	ADPKD mice, IRI-induced AKI mice, UUO-induced CKD mice	↓ Ras/MAPK, TGF-β/Smad, IL-6, COX-2, and iNOS ↓ TLR4/MyD88/NF-κB, and caspase-3	[159,160,161]
*Glycyrrhiza uralensis* Fisch.	Glycyrrhizinic acid, glycyrrhizic acid	DN mice, TAC/PC-induced AKI mice	↓ ROS, IL-1β, IL-6, TNF-α, CCR2 ↑ AMPK/SIRT1/PGC-1α Regulates autophagy	[162,163,164,165,166,167]
**Alkaloids**				
*Ligusticum sinense* Chuanxiong ^S^	Tetramethylpyrazine	PC/IRI-induced AKI rats, DN rats	↓ CCL2/CCR2, ROS, NLRP3, TNF-α ↑ AKT, Bcl-2	[168,169,170,171,172]
*Leonurus japonicus* Houtt. ^S^	Leonurine	DDP/LPS/vancomycin-induced AKI mice, UUO-induced CKD mice	↑ Nrf2 ↓ TLR4/MyD88/NF-κB, TNF-α, IL-1β, TGF-β/Smad3	[173,174,175,176,177]
*Coptis chinensis*	Berberine	IRI/DOX/DDP/MTX/gentamicin-induced AKI, DN mice, and UUO-induced CKD mice, UAN mice	↓ IL-6, IL-10, TGF-β/Smad3, mitochondrial stress, and ER stress ↑ Nrf2, MDA, SOD, CAT, GSH, and Bcl-2 Regulating gut microbiota	[178,179,180,181,182,183,184,185,186,187,188,189,190]
*Trigonella foenum-graecum* L. ^S^	Trigonelline	ON mice, DN mice	↓ EMT, ROS, α-SMA ↑ AMPK pathway	[191,192,193,194,195,196]
*Piper longum* L. ^S^	Piperlonguminine	UUO-induced CKD mice	↓ TRPC6	[197]
*Sophora japonica* L.	Matrine, oxymatrine	IRI/DDP/gentamicin-induced AKI mice, UUO-induced CKD mice	↓ IL-6, IL-10, TGF-β/Smad3	[198,199,200]
*Nelumbo nucifera* Gaertn.	Liensinine, Isoliensinine	IRI-induced AKI rats, LPS-induced AKI mice	↓ TGF-β1/Smad3	[201,202]
*Nelumbo nucifera* Gaertn.	Neferine, nuciferine	UAN rats, LPS/IRI-induced AKI mice, DN mice	↓ TLR4/MyD88/NF-κB, IL-1β ↑ Bcl-2	[203,204,205,206,207,208,209,210]
**Others**				
*Curcuma longa* L.	Curcumin	LN mice, patulin-induced AKI mice, CKD patients, UAN rats	↑ Nrf2/FOXO-3a, GSH ↓ PI3K/AKT/NF-κB, TGF-β, ROS Regulating gut microbiota	[211,212,213,214,215]
*Rheum palmatum* L.	Emodin, aloe emodin, rhein	UUO-induced CKD mice/rats, DN mice	↓ IL-1β, TGF-β, PERK-eIF2α Regulating gut microbiota	[216,217,218,219,220]
*Eugenia caryophyllata* Thunb.	Eugenol	IRI/patulin-induced AKI mice	↓ TGF-β ↑ Nrf2	[221,222]
*Sesamum indicum* L.	Sesamin	CKD mice, DDP/LPS-induced AKI mice	↑ GSH, CAT, and SOD Regulating gut microbiota	[223,224,225,226]
*Ligusticum sinense* Chuanxiong ^S^	Ferulic acid	UAN mice	↓ TLR4/NF-κB Regulating gut microbiota	[227]
*Lonicera japonica* Thunb.	Chlorogenic acid	UAN mice	↓ IL-1β, TNF-α, IL-6 Regulating gut microbiota	[228,229,230]

^S^: These botanicals are medical herbs that could be added to dietary supplements in the index of Table S1; ↑:upregulating or promoting; ↓: downregulating or inhibiting; PI3K: Phosphoinositide 3-kinase; AKT: Protein kinase B; Nrf2: Nuclear factor erythroid 2-related factor 2; HO-1: Heme oxygenase 1; ICAM: Intercellular adhesion molecule; VCAM: Vascular cell adhesion molecule; NLRP3: NOD-, LRR-, and pyrin domain-containing protein 3; Bax: BCL2-associated X; Bcl-2: B-cell lymphoma 2; MAPK: Mitogen-activated protein kinase; AMPK: Adenosine monophosphate-activated protein kinase; NF-κB: Nuclear factor kappa-B; mTOR: Mammalian target of rapamycin; cAMP: Cyclic adenosine monophosphate; PKA: Protein kinase A; CREB: cAMP-response element binding protein; TLR: Toll-like receptor; RAGE: Receptor for AGEs; AGEs: Advanced glycation end products; RhoA: Ras homolog family member A; Ras: Rat sarcoma; ROCK: Rho-associated coiled-coil-containing protein kinase; TGF: Transforming growth factor; iNOS: Inducible nitric oxide synthase; COX: Cyclooxygenase; CCR: Chemokine (C-C motif) receptor; CCL/MCP: Chemokine (C-C motif) ligand; PGC: Peroxisome proliferator-activated receptor-gamma coactivator; FXR: Farnesoid X receptor; α-SMA: α-Smooth muscle actin; ROS: Reactive oxygen species; GSK-3β: Glycogen synthase kinase 3 beta; SOD: Superoxide dismutase; GSH: Glutathione; CAT: Catalase; HIF: Hypoxia-inducible factor; NOX: NADPH oxidase; IL: Interleukin; TNF: Tumor necrosis factor; FOXO: Forkhead box O; ERK: Extracellular regulated protein kinase; MMP: Matrix metalloproteinase; ER: Endoplasmic reticulum; PERK: Protein kinase R-like endoplasmic reticulum kinase; eIF2α: Phosphorylation of eukaryotic initiation factor-2α; ATF4: Activating transcription factor 4; SIRT1: Silent information regulator 1; TRPC: Transient receptor potential canonical; SCFAs: Short-chain fatty acids; CKD: Chronic kidney disease; IRI: Ischemia/reperfusion injury; ADPKD: Autosomal dominant polycystic kidney disease; UUO: Unilateral ureteral obstruction; UAN: Uric acid nephropathy; CLP: Cecum ligation puncture; DOX: Doxorubicin; DDP: Cisplatin; LPS: Lipopolysaccharide; EG: Ethylene glycol; LN: Lupus nephritis; Ang II: Angiotensin II; HMCs: Human mesangial cells; HK-2 cells: Human kidney-2 cells; Fru: Fructose; TAC: Tacrolimus; PC: Post-contrast; MTX: Methotrexate; ON: Oxalate nephropathy.

## Data Availability

No new data were created or analyzed in this study. Data sharing is not applicable to this article.

## Supplementary Materials

The following supporting information can be downloaded at: https://www.mdpi.com/article/10.3390/nu16203530/s1, Table S1: Botanicals that are allowed to be added to dietary supplements in China.

## References

[1] Wu H., Huang J. (2018). Drug-Induced Nephrotoxicity: Pathogenic Mechanisms, Biomarkers and Prevention Strategies. Curr. Drug Metab..

[2] Mody H., Ramakrishnan V., Chaar M., Lezeau J., Rump A., Taha K., Lesko L., Ait-Oudhia S. (2020). A Review on Drug-Induced Nephrotoxicity: Pathophysiological Mechanisms, Drug Classes, Clinical Management, and Recent Advances in Mathematical Modeling and Simulation Approaches. Clin. Pharmacol. Drug Dev..

[3] Schetz M., Dasta J., Goldstein S., Golper T. (2005). Drug-Induced Acute Kidney Injury. Curr. Opin. Crit. Care.

[4] Yang T., Richards E.M., Pepine C.J., Raizada M.K. (2018). The Gut Microbiota and the Brain–Gut–Kidney Axis in Hypertension and Chronic Kidney Disease. Nat. Rev. Nephrol..

[5] Gaitonde D.Y., Cook D.L., Rivera I.M. (2017). Chronic Kidney Disease: Detection and Evaluation. Am. Fam. Physician.

[6] Tain Y.L., Chang C.I., Hou C.Y., Chang-Chien G.P., Lin S.F., Hsu C.N. (2024). Resveratrol Propionate Ester Supplement Exerts Antihypertensive Effect in Juvenile Rats Exposed to an Adenine Diet Via Gut Microbiota Modulation. Nutrients.

[7] Bellomo F., Pugliese S., Cairoli S., Krohn P., De Stefanis C., Raso R., Rega L.R., Taranta A., De Leo E., Ciolfi A. (2024). Ketogenic Diet and Progression of Kidney Disease in Animal Models of Nephropathic Cystinosis. J. Am. Soc. Nephrol..

[8] Yang C., Wang T., Chen J., He J., Li Y., Chen C., Lu G., Chen W. (2021). Traditional Chinese Medicine Formulas Alleviate Acute Pancreatitis: Pharmacological Activities and Mechanisms. Pancreas.

[9] Wang Y., Li M., Liang Y., Yang Y., Liu Z., Yao K., Chen Z., Zhai S. (2017). Chinese Herbal Medicine for the Treatment of Depression: Applications, Efficacies and Mechanisms. Curr. Pharm. Des..

[10] Chan H.H.L., Ng T. (2020). Traditional Chinese Medicine (Tcm) and Allergic Diseases. Curr. Allergy Asthma Rep..

[11] Wojcikowski K., Wohlmuth H., Johnson D.W., Rolfe M., Gobe G. (2009). An In Vitro Investigation of Herbs Traditionally Used for Kidney and Urinary System Disorders: Potential Therapeutic and Toxic Effects. Nephrology.

[12] Li C., Huang H., Wang R., Zhang C., Huang S., Wu J., Mo P., Yu H., Li S., Chen J. (2023). Formula Restores Iron Metabolism from Dysregulation in Anemic Rats with Adenine-Induced Nephropathy. J. Ethnopharmacol..

[13] Ll J., Yu D.R., Chen H.Y., Zhu C.F., Cheng X.X., Wang Y.H., Ni J., Wang X.J., Jinag F. (2017). Long-Term Effect of the Treatment of Iga Nephropathy by Tonifying Shen, Activating Blood Stasis, Dispelling Wind-Dampness Combined with Western Medicine. Zhongguo Zhong Xi Yi Jie He Za Zhi.

[14] Peng M., Cai P., Ma H., Meng H., Xu Y., Zhang X., Si G. (2014). Chinese Herbal Medicine Shenqi Detoxification Granule Inhibits Fibrosis in Adenine Induced Chronic Renal Failure Rats. Afr. J. Tradit. Complement. Altern. Med..

[15] (2002). Notice of the National Health Commission on Further Regulating the Management of Health Food Raw Materials (No. 51 of 2002).

[16] Wan F., Ma F., Wu J., Qiao X., Chen M., Li W., Ma L. (2022). Effect of *Lycium barbarum* Polysaccharide on Decreasing Serum Amyloid A3 Expression through Inhibiting Nf-Κb Activation in a Mouse Model of Diabetic Nephropathy. Anal. Cell. Pathol..

[17] Li W., Yu L., Fu B., Chu J., Chen C., Li X., Ma J., Tang W. (2022). Protective Effects of *Polygonatum kingianum* Polysaccharides and Aqueous Extract on Uranium-Induced Toxicity in Human Kidney (Hk-2) Cells. Int. J. Biol. Macromol..

[18] Yue L., Wang W., Wang Y., Du T., Shen W., Tang H., Wang Y., Yin H. (2016). *Bletilla striata* Polysaccharide Inhibits Angiotensin Ii-Induced Ros and Inflammation Via Nox4 and Tlr2 Pathways. Int. J. Biol. Macromol..

[19] Ma Q., Xu Y., Tang L., Yang X., Chen Z., Wei Y., Shao X., Shao X., Xin Z., Cai B. (2020). Astragalus Polysaccharide Attenuates Cisplatin-Induced Acute Kidney Injury by Suppressing Oxidative Damage and Mitochondrial Dysfunction. BioMed Res. Int..

[20] Zhao K., Wu X., Han G., Sun L., Zheng C., Hou H., Xu B.B., El-Bahy Z.M., Qian C., Kallel M. (2024). *Phyllostachys nigra* (Lodd. Ex Lindl.) Derived Polysaccharide with Enhanced Glycolipid Metabolism Regulation and Mice Gut Microbiome. Int. J. Biol. Macromol..

[21] Dwyer J.T., Coates P.M., Smith M.J. (2018). Dietary Supplements: Regulatory Challenges and Research Resources. Nutrients.

[22] Nicastro H.L., Vorkoper S., Sterling R., Korn A.R., Brown A.G.M., Maruvada P., Oh A.Y. (2023). Opportunities to Advance Implementation Science and Nutrition Research: A Commentary on the Strategic Plan for Nih Nutrition Research. Transl. Behav. Med..

[23] Bilia A.R. (2015). Herbal Medicinal Products Versus Botanical-Food Supplements in the European Market: State of Art and Perspectives. Nat. Prod. Commun..

[24] Luciano R.L., Perazella M.A. (2015). Aristolochic Acid Nephropathy: Epidemiology, Clinical Presentation, and Treatment. Drug Saf..

[25] Ban T.H., Min J.W., Seo C., Kim D.R., Lee Y.H., Chung B.H., Jeong K.H., Lee J.W., Kim B.S., Lee S.H. (2018). Update of Aristolochic Acid Nephropathy in Korea. Korean J. Intern. Med..

[26] Dugo M., Gatto R., Zagatti R., Gatti P., Cascone C. (2010). Herbal Remedies: Nephrotoxicity and Drug Interactions. G. Ital. Nefrol..

[27] Yang H., Zhao Y., Ren B., Wu Y., Qiu Z., Cheng Y., Qiu B. (2024). Poria Acid Inhibit the Growth and Metastasis of Renal Cell Carcinoma by Inhibiting the Pi3k/Akt/Nf-Κb Signaling Pathway. Heliyon.

[28] Lu M., Yin J., Xu T., Dai X., Liu T., Zhang Y., Wang S., Liu Y., Shi H., Zhang Y. (2024). Fuling-Zexie Formula Attenuates Hyperuricemia-Induced Nephropathy and Inhibits Jak2/Stat3 Signaling and Nlrp3 Inflammasome Activation in Mice. J. Ethnopharmacol..

[29] Food Safety Standards and Evaluation Division (2019). Notice on Six New Substances Including Angelica Sinensis and Other Substances Which Are Both Food and Chinese Herbal Medicine According to Tradition (No. 8 of 2019).

[30] Food Safety Standards and Evaluation Division (2023). Notice on Nine New Substances Including Codonopsis Pilosula and Other Substances Which Are Both Food and Chinese Herbal Medicine According to Tradition (No. 9 of 2023).

[31] Food Safety Standards and Evaluation Division (2021). Regulations on Catalog of Substances That Are Traditionally Used as Both Food and Herbal Medicine.

[32] Vettorazzi A., de Cerain A.L., Sanz-Serrano J., Gil A.G., Azqueta A. (2020). European Regulatory Framework and Safety Assessment of Food-Related Bioactive Compounds. Nutrients.

[33] Bailey R.L. (2018). Current Regulatory Guidelines and Resources to Support Research of Dietary Supplements in the United States. Crit. Rev. Food Sci. Nutr..

[34] US Department of Health and Human Services, National Institutes of Health, Office of Dietary Supplements (2013). Dietary Supplement Label Database (Dsld).

[35] Liu Y., Luo J., Peng L., Zhang Q., Rong X., Luo Y., Li J. (2024). Flavonoids: Potential Therapeutic Agents for Cardiovascular Disease. Heliyon.

[36] Frydman A., Weisshaus O., Bar-Peled M., Huhman D.V., Sumner L.W., Marin F.R., Lewinsohn E., Fluhr R., Gressel J., Eyal Y. (2004). Citrus Fruit Bitter Flavors: Isolation and Functional Characterization of the Gene Cm1,2rhat Encoding a 1,2 Rhamnosyltransferase, a Key Enzyme in the Biosynthesis of the Bitter Flavonoids of Citrus. Plant J..

[37] Zhang L., Wang X., Chang L., Ren Y., Sui M., Fu Y., Zhang L., Hao L. (2024). Quercetin Improves Diabetic Kidney Disease by Inhibiting Ferroptosis and Regulating the Nrf2 in Streptozotocin-Induced Diabetic Rats. Ren. Fail..

[38] Guo X., Wen S., Wang J., Zeng X., Yu H., Chen Y., Zhu X., Xu L. (2024). Senolytic Combination of Dasatinib and Quercetin Attenuates Renal Damage in Diabetic Kidney Disease. Phytomedicine.

[39] Zeng Y.F., Li J.Y., Wei X.Y., Ma S.Q., Wang Q.G., Qi Z., Duan Z.C., Tan L., Tang H. (2023). Preclinical Evidence of Reno-Protective Effect of Quercetin on Acute Kidney Injury: A Meta-Analysis of Animal Studies. Front. Pharmacol..

[40] Xu Z., Wang X., Kuang W., Wang S., Zhao Y. (2023). Kaempferol Improves Acute Kidney Injury Via Inhibition of Macrophage Infiltration in Septic Mice. Biosci. Rep..

[41] Yuan N., Diao J., Dong J., Yan Y., Chen Y., Yan S., Liu C., He Z., He J., Zhang C. (2024). Targeting Rock1 in Diabetic Kidney Disease: Unraveling Mesangial Fibrosis Mechanisms and Introducing Myricetin as a Novel Antagonist. Biomed. Pharmacother..

[42] Yang Z.J., Wang H.R., Wang Y.I., Zhai Z.H., Wang L.W., Li L., Zhang C., Tang L. (2019). Myricetin Attenuated Diabetes-Associated Kidney Injuries and Dysfunction Via Regulating Nuclear Factor (Erythroid Derived 2)-Like 2 and Nuclear Factor-Κb Signaling. Front. Pharmacol..

[43] Liu P., Tang L., Li G., Wu X., Hu F., Peng W. (2024). Association between Consumption of Flavonol and Its Subclasses and Chronic Kidney Disease in Us Adults: An Analysis Based on National Health and Nutrition Examination Survey Data from 2007–2008, 2009–2010, and 2017–2018. Front. Nutr..

[44] Jian J., Li Y.-Q., Han R.-Y., Zhong X., Xie K.-H., Yan Y., Wang L., Tan R.-Z. (2024). Isorhamnetin Ameliorates Cisplatin-Induced Acute Kidney Injury in Mice by Activating Slpi-Mediated Anti-Inflammatory Effect in Macrophage. Immunopharmacol. Immunotoxicol..

[45] Wang B., Yang L.N., Yang L.T., Liang Y., Guo F., Fu P., Ma L. (2024). Fisetin Ameliorates Fibrotic Kidney Disease in Mice Via Inhibiting Acsl4-Mediated Tubular Ferroptosis. Acta Pharmacol. Sin..

[46] Zou T.F., Liu Z.G., Cao P.C., Zheng S.H., Guo W.T., Wang T.X., Chen Y.L., Duan Y.J., Li Q.S., Liao C.Z. (2023). Fisetin Treatment Alleviates Kidney Injury in Mice with Diabetes-Exacerbated Atherosclerosis through Inhibiting Cd36/Fibrosis Pathway. Acta Pharmacol. Sin..

[47] Zhang D., Liu S., Jiang H., Liu S., Kong F. (2024). Dia Proteomics Analysis Reveals the Mechanism of Folic Acid-Induced Acute Kidney Injury and the Effects of Icariin. Chem. Biol. Interact..

[48] Zhao Y., Yang W., Zhang X., Lv C., Lu J. (2023). Icariin, the Main Prenylflavonoid of Epimedii Folium, Ameliorated Chronic Kidney Disease by Modulating Energy Metabolism Via Ampk Activation. J. Ethnopharmacol..

[49] Owumi S.E., Kazeem A.I., Wu B., Ishokare L.O., Arunsi U.O., Oyelere A.K. (2022). Apigeninidin-Rich *Sorghum bicolor* (L. Moench) Extracts Suppress A549 Cells Proliferation and Ameliorate Toxicity of Aflatoxin B1-Mediated Liver and Kidney Derangement in Rats. Sci. Rep..

[50] Amorim J.M., de Souza L.C.R., de Souza R.A.L., da Silva Filha R., de Oliveira Silva J., de Almeida Araújo S., Tagliti C.A., Simões E.S.A.C., Castilho R.O. (2022). Costus Spiralis Extract Restores Kidney Function in Cisplatin-Induced Nephrotoxicity Model: Ethnopharmacological Use, Chemical and Toxicological Investigation. J. Ethnopharmacol..

[51] Wang X., Kim C.S., Adams B.C., Wilkinson R., Hill M.M., Shah A.K., Mohamed A., Dutt M., Ng M.S.Y., Ungerer J.P.J. (2024). Human Proximal Tubular Epithelial Cell-Derived Small Extracellular Vesicles Mediate Synchronized Tubular Ferroptosis in Hypoxic Kidney Injury. Redox Biol..

[52] Guo S., Zhou L., Liu X., Gao L., Li Y., Wu Y. (2024). Baicalein Alleviates Cisplatin-Induced Acute Kidney Injury by Inhibiting Alox12-Dependent Ferroptosis. Phytomedicine.

[53] Hu H., Li W., Hao Y., Peng Z., Zou Z., Liang W. (2024). Baicalin Ameliorates Renal Fibrosis by Upregulating Cpt1α-Mediated Fatty Acid Oxidation in Diabetic Kidney Disease. Phytomedicine.

[54] Miguel V., Rey-Serra C., Tituaña J., Sirera B., Alcalde-Estévez E., Herrero J.I., Ranz I., Fernández L., Castillo C., Sevilla L. (2023). Enhanced Fatty Acid Oxidation through Metformin and Baicalin as Therapy for COVID-19 and Associated Inflammatory States in Lung and Kidney. Redox Biol..

[55] Liu B., Deng Q., Zhang L., Zhu W. (2020). Nobiletin Alleviates Ischemia/Reperfusion Injury in the Kidney by Activating Pi3k/Akt Pathway. Mol. Med. Rep..

[56] Song J., Wang H., Sheng J., Zhang W., Lei J., Gan W., Cai F., Yang Y. (2023). Vitexin Attenuates Chronic Kidney Disease by Inhibiting Renal Tubular Epithelial Cell Ferroptosis Via Nrf2 Activation. Mol. Med..

[57] Ding T., Zhao T., Li Y., Liu Z., Ding J., Ji B., Wang Y., Guo Z. (2021). Vitexin Exerts Protective Effects against Calcium Oxalate Crystal-Induced Kidney Pyroptosis In Vivo and In Vitro. Phytomedicine.

[58] Yang Q., Qian L., He S., Zhang C. (2024). Hesperidin Alleviates Zinc-Induced Nephrotoxicity Via the Gut-Kidney Axis in Swine. Front. Cell Infect. Microbiol..

[59] Chen J., Fan X., Chen J., Luo X., Huang X., Zhou Z., He Y., Feng S., Jiao Y., Wang R. (2024). Effects of Hesperidin on the Histological Structure, Oxidative Stress, and Apoptosis in the Liver and Kidney Induced by Nicl(2). Front. Vet. Sci..

[60] Yang A.Y., Choi H.J., Kim K., Leem J. (2023). Antioxidant, Antiapoptotic, and Anti-Inflammatory Effects of Hesperetin in a Mouse Model of Lipopolysaccharide-Induced Acute Kidney Injury. Molecules.

[61] Chen X., Wei W., Li Y., Huang J., Ci X. (2019). Hesperetin Relieves Cisplatin-Induced Acute Kidney Injury by Mitigating Oxidative Stress, Inflammation and Apoptosis. Chem. Biol. Interact..

[62] Chen C., Feng F., Qi M., Chen Q., Tang W., Diao H., Hu Z., Qiu Y., Li Z., Chu Y. (2024). Dietary Citrus Flavonoids Improved Growth Performance and Intestinal Microbiota of Weaned Piglets Via Immune Function Mediated by Tlr2/Nf-Κb Signaling Pathway. J. Agric. Food Chem..

[63] Chang Y.W., Chen Y.L., Park S.H., Yap E.E.S., Sung W.C. (2024). Characterization of Functional Ingredients Extracted with Ethanol Solvents from Ponkan (*Citrus reticulata*) by-Products Using the Microwave Vacuum Drying Method Combined with Ultrasound-Assisted Extraction. Foods.

[64] Chen Y., Pan H., Hao S., Pan D., Wang G., Yu W. (2021). Evaluation of Phenolic Composition and Antioxidant Properties of Different Varieties of Chinese Citrus. Food Chem..

[65] Kamel K.M., El-Raouf O.M.A., Metwally S.A., El-Latif H.A.A., El-sayed M.E. (2014). Hesperidin and Rutin, Antioxidant Citrus Flavonoids, Attenuate Cisplatin-Induced Nephrotoxicity in Rats. J. Biochem. Mol. Toxicol..

[66] Jung H.A., Jung M.J., Kim J.Y., Chung H.Y., Choi J.S. (2003). Inhibitory Activity of Flavonoids from Prunus Davidiana and Other Flavonoids on Total Ros and Hydroxyl Radical Generation. Arch. Pharm. Res..

[67] Mbara K.C., Fotsing M.C.D., Ndinteh D.T., Mbeb C.N., Nwagwu C.S., Khan R., Mokhetho K.C., Baijnath H., Nlooto M., Mokhele S. (2024). Endoplasmic Reticulum Stress in Pancreatic Β-Cell Dysfunction: The Potential Therapeutic Role of Dietary Flavonoids. Curr. Res. Pharmacol. Drug Discov..

[68] Ramos F.M.M., Ribeiro C.B., Cesar T.B., Milenkovic D., Cabral L., Noronha M.F., Sivieri K. (2023). Lemon Flavonoids Nutraceutical (Eriomin^®^) Attenuates Prediabetes Intestinal Dysbiosis: A Double-Blind Randomized Controlled Trial. Food Sci. Nutr..

[69] Bellavite P. (2023). Neuroprotective Potentials of Flavonoids: Experimental Studies and Mechanisms of Action. Antioxidants.

[70] Pabich M., Materska M. (2019). Biological Effect of Soy Isoflavones in the Prevention of Civilization Diseases. Nutrients.

[71] Misiakiewicz-Has K., Maciejewska-Markiewicz D., Szypulska-Koziarska D., Kolasa A., Wiszniewska B. (2024). The Influence of Soy Isoflavones and Soy Isoflavones with Inulin on Kidney Morphology, Fatty Acids, and Associated Parameters in Rats with and without Induced Diabetes Type 2. Int. J. Mol. Sci..

[72] Hu Z., Chen D., Yan P., Zheng F., Zhu H., Yuan Z., Yang X., Zuo Y., Chen C., Lu H. (2024). Puerarin Suppresses Macrophage M1 Polarization to Alleviate Renal Inflammatory Injury through Antagonizing Tlr4/Myd88-Mediated Nf-Κb P65 and Jnk/Foxo1 Activation. Phytomedicine.

[73] Zhang S.S., Zhang N.N., Guo S., Liu S.J., Hou Y.F., Li S., Ho C.T., Bai N.S. (2022). Glycosides and Flavonoids from the Extract of Pueraria Thomsonii Benth Leaf Alleviate Type 2 Diabetes in High-Fat Diet Plus Streptozotocin-Induced Mice by Modulating the Gut Microbiota. Food Funct..

[74] Zhou Y., Lu W., Huang K., Gan F. (2024). Ferroptosis Is Involved in Quercetin-Mediated Alleviation of Ochratoxin a-Induced Kidney Damage. Food Chem. Toxicol..

[75] Liu F., Feng Q., Yang M., Yang Y., Nie J., Wang S. (2024). Quercetin Prevented Diabetic Nephropathy by Inhibiting Renal Tubular Epithelial Cell Apoptosis Via the Pi3k/Akt Pathway. Phytother. Res..

[76] Sheng H., Zhang D., Zhang J., Zhang Y., Lu Z., Mao W., Liu X., Zhang L. (2022). Kaempferol Attenuated Diabetic Nephropathy by Reducing Apoptosis and Promoting Autophagy through Ampk/Mtor Pathways. Front. Med..

[77] Wu Q., Chen J., Zheng X., Song J., Yin L., Guo H., Chen Q., Liu Y., Ma Q., Zhang H. (2023). Kaempferol Attenuates Doxorubicin-Induced Renal Tubular Injury by Inhibiting Ros/Ask1-Mediated Activation of the Mapk Signaling Pathway. Biomed. Pharmacother..

[78] Guan Y., Quan D., Chen K., Kang L., Yang D., Wu H., Yan M., Wu S., Lv L., Zhang G. (2023). Kaempferol Inhibits Renal Fibrosis by Suppression of the Sonic Hedgehog Signaling Pathway. Phytomedicine.

[79] Yang X., Zhang P., Jiang J., Almoallim H.S., Alharbi S.A., Li Y. (2023). Myricetin Attenuates Ethylene Glycol-Induced Nephrolithiasis in Rats Via Mitigating Oxidative Stress and Inflammatory Markers. Appl. Biochem. Biotechnol..

[80] Liu J., Xie L., Zhai H., Wang D., Li X., Wang Y., Song M., Xu C. (2024). Exploration of the Protective Mechanisms of Icariin against Cisplatin-Induced Renal Cell Damage in Canines. Front. Vet. Sci..

[81] Ding N., Sun S., Zhou S., Lv Z., Wang R. (2024). Icariin Alleviates Renal Inflammation and Tubulointerstitial Fibrosis Via Nrf2-Mediated Attenuation of Mitochondrial Damage. Cell Biochem. Funct..

[82] Duan S., Ding Z., Liu C., Wang X., Dai E. (2024). Icariin Suppresses Nephrotic Syndrome by Inhibiting Pyroptosis and Epithelial-to-Mesenchymal Transition. PLoS ONE.

[83] Wu Q., Li W., Zhao J., Sun W., Yang Q., Chen C., Xia P., Zhu J., Zhou Y., Huang G. (2021). Apigenin Ameliorates Doxorubicin-Induced Renal Injury Via Inhibition of Oxidative Stress and Inflammation. Biomed. Pharmacother..

[84] Li Y., Zhao Z., Luo J., Jiang Y., Li L., Chen Y., Zhang L., Huang Q., Cao Y., Zhou P. (2021). Apigenin Ameliorates Hyperuricemic Nephropathy by Inhibiting Urat1 and Glut9 and Relieving Renal Fibrosis Via the Wnt/Β-Catenin Pathway. Phytomedicine.

[85] Zamani F., Samiei F., Mousavi Z., Azari M.R., Seydi E., Pourahmad J. (2021). Apigenin Ameliorates Oxidative Stress and Mitochondrial Damage Induced by Multiwall Carbon Nanotubes in Rat Kidney Mitochondria. J. Biochem. Mol. Toxicol..

[86] Wang T., Zhang Z., Xie M., Li S., Zhang J., Zhou J. (2022). Apigenin Attenuates Mesoporous Silica Nanoparticles-Induced Nephrotoxicity by Activating Foxo3a. Biol. Trace Elem. Res..

[87] Azimi A., Eidi A., Mortazavi P., Rohani A.H. (2021). Protective Effect of Apigenin on Ethylene Glycol-Induced Urolithiasis Via Attenuating Oxidative Stress and Inflammatory Parameters in Adult Male Wistar Rats. Life Sci..

[88] Zhang K., Li J., Dong W., Huang Q., Wang X., Deng K., Ali W., Song R., Zou H., Ran D. (2024). Luteolin Alleviates Cadmium-Induced Kidney Injury by Inhibiting Oxidative DNA Damage and Repairing Autophagic Flux Blockade in Chickens. Antioxidants.

[89] Xu X., Yu Z., Han B., Li S., Sun Y., Du Y., Wang Z., Gao D., Zhang Z. (2021). Luteolin Alleviates Inorganic Mercury-Induced Kidney Injury Via Activation of the Ampk/Mtor Autophagy Pathway. J. Inorg. Biochem..

[90] Ding T., Yi T., Li Y., Zhang W., Wang X., Liu J., Fan Y., Ji J., Xu L. (2023). Luteolin Attenuates Lupus Nephritis by Regulating Macrophage Oxidative Stress Via Hif-1α Pathway. Eur. J. Pharmacol..

[91] Li F., Wei R., Huang M., Chen J., Li P., Ma Y., Chen X. (2022). Luteolin Can Ameliorate Renal Interstitial Fibrosis-Induced Renal Anaemia through the Sirt1/Foxo3 Pathway. Food Funct..

[92] Awoyomi O.V., Adeoye Y.D., Oyagbemi A.A., Ajibade T.O., Asenuga E.R., Gbadamosi I.T., Ogunpolu B.S., Falayi O.O., Hassan F.O., Omobowale T.O. (2021). Luteolin Mitigates Potassium Dichromate-Induced Nephrotoxicity, Cardiotoxicity and Genotoxicity through Modulation of Kim-1/Nrf2 Signaling Pathways. Environ. Toxicol..

[93] Zhu Q., Yang S., Wei C., Lu G., Lee K., He J.C., Liu R., Zhong Y. (2022). Puerarin Attenuates Diabetic Kidney Injury through Interaction with Guanidine Nucleotide-Binding Protein Gi Subunit Alpha-1 (Gnai1) Subunit. J. Cell Mol. Med..

[94] Song Q., Jian W., Zhang Y., Li Q., Zhao Y., Liu R., Zeng Y., Zhang F., Duan J. (2024). Puerarin Attenuates Iron Overload-Induced Ferroptosis in Retina through a Nrf2-Mediated Mechanism. Mol. Nutr. Food Res..

[95] Jing G.H., Liu Y.D., Liu J.N., Jin Y.S., Yu S.L., An R.H. (2022). Puerarin Prevents Calcium Oxalate Crystal-Induced Renal Epithelial Cell Autophagy by Activating the Sirt1-Mediated Signaling Pathway. Urolithiasis.

[96] Sun J., Wei S., Zhang Y., Li J. (2021). Protective Effects of Astragalus Polysaccharide on Sepsis-Induced Acute Kidney Injury. Anal. Cell. Pathol..

[97] Li J., Zhao J., Chai Y., Li W., Liu X., Chen Y. (2022). Astragalus Polysaccharide Protects Sepsis Model Rats after Cecum Ligation and Puncture. Front. Bioeng. Biotechnol..

[98] Wang Y., Liu D., Chen S., Wang Y., Jiang H., Yin H. (2014). A New Glucomannan from Bletilla Striata: Structural and Anti-Fibrosis Effects. Fitoterapia.

[99] Pan Y., Zhang Y., Li J., Zhang Z., He Y., Zhao Q., Yang H., Zhou P. (2023). A Proteoglycan Isolated from Ganoderma Lucidum Attenuates Diabetic Kidney Disease by Inhibiting Oxidative Stress-Induced Renal Fibrosis Both In Vitro and In Vivo. J. Ethnopharmacol..

[100] Zhong D., Wang H., Liu M., Li X., Huang M., Zhou H., Lin S., Lin Z., Yang B. (2015). Ganoderma Lucidum Polysaccharide Peptide Prevents Renal Ischemia Reperfusion Injury Via Counteracting Oxidative Stress. Sci. Rep..

[101] Xie W., Huang Y.-Y., Chen H.-G., Zhou X. (2021). Study on the Efficacy and Mechanism of Lycium Barbarum Polysaccharide against Lead-Induced Renal Injury in Mice. Nutrients.

[102] Chen J., Wang W., Zhang Q., Li F., Lei T., Luo D., Zhou H., Yang B. (2013). Low Molecular Weight Fucoidan against Renal Ischemia–Reperfusion Injury Via Inhibition of the Mapk Signaling Pathway. PLoS ONE.

[103] Li X.-Y., Chen H.-R., Zha X.-Q., Chen S., Pan L.-H., Li Q.-M., Luo J.-P. (2020). Prevention and Possible Mechanism of a Purified Laminaria Japonica Polysaccharide on Adriamycin-Induced Acute Kidney Injury in Mice. Int. J. Biol. Macromol..

[104] Wang Y., Sun Y., Shao F., Zhang B., Wang Z., Li X. (2021). Low Molecular Weight Fucoidan Can Inhibit the Fibrosis of Diabetic Kidneys by Regulating the Kidney Lipid Metabolism. J. Diabetes Res..

[105] Chen J., Cui W., Zhang Q., Jia Y., Sun Y., Weng L., Luo D., Zhou H., Yang B. (2015). Low Molecular Weight Fucoidan Ameliorates Diabetic Nephropathy Via Inhibiting Epithelial-Mesenchymal Transition and Fibrotic Processes. Am. J. Transl. Res..

[106] Li X.-Y., Chen H.-R., Kuang D.-D., Pan L.-H., Li Q.-M., Luo J.-P., Zha X.-Q. (2023). Laminaria Japonica Polysaccharide Attenuates Podocyte Epithelial-Mesenchymal Transformation Via Tgf-Β1-Mediated Smad3 and P38mapk Pathways. Int. J. Biol. Macromol..

[107] Wang J., Zhang Q., Li S., Chen Z., Tan J., Yao J., Duan D. (2020). Low Molecular Weight Fucoidan Alleviates Diabetic Nephropathy by Binding Fibronectin and Inhibiting Ecm-Receptor Interaction in Human Renal Mesangial Cells. Int. J. Biol. Macromol..

[108] Li X., Li X., Zhang Q., Zhao T. (2017). Low Molecular Weight Fucoidan and Its Fractions Inhibit Renal Epithelial Mesenchymal Transition Induced by Tgf-Β1 or Fgf-2. Int. J. Biol. Macromol..

[109] Wei X.-M., Jiang S., Li S.-S., Sun Y.-S., Wang S.-H., Liu W.-C., Wang Z., Wang Y.-P., Zhang R., Li W. (2021). Endoplasmic Reticulum Stress-Activated Perk-Eif2α-Atf4 Signaling Pathway Is Involved in the Ameliorative Effects of Ginseng Polysaccharides against Cisplatin-Induced Nephrotoxicity in Mice. ACS Omega.

[110] Lian Y., Zhu M., Chen J., Yang B., Lv Q., Wang L., Guo S., Tan X., Li C., Bu W. (2021). Characterization of a Novel Polysaccharide from Moutan Cortex and Its Ameliorative Effect on Ages-Induced Diabetic Nephropathy. Int. J. Biol. Macromol..

[111] Zhang M., Yang L., Zhu M., Yang B., Yang Y., Jia X., Feng L. (2022). Moutan Cortex Polysaccharide Ameliorates Diabetic Kidney Disease Via Modulating Gut Microbiota Dynamically in Rats. Int. J. Biol. Macromol..

[112] Zhao H., Xu J., Wang R., Tang W., Kong L., Wang W., Wang L., Zhang Y., Ma W. (2021). Plantaginis Semen Polysaccharides Ameliorate Renal Damage through Regulating Nlrp3 Inflammasome in Gouty Nephropathy Rats. Food Funct..

[113] Huang C., Yu J., Da J., Dong R., Dai L., Yang Y., Deng Y., Yuan J. (2023). Dendrobium Officinale Kimura & Migo Polysaccharide Inhibits Hyperglycaemia-Induced Kidney Fibrosis Via the Mirna-34a-5p/Sirt1 Signalling Pathway. J. Ethnopharmacol..

[114] Wang X., Liu W., Jin G., Wu Z., Zhang D., Bao Y., Shi W. (2022). Salvia Miltiorrhiza Polysaccharides Alleviates Florfenicol Induced Kidney Injury in Chicks Via Inhibiting Oxidative Stress and Apoptosis. Ecotoxicol. Environ. Saf..

[115] Zhang J., Bi R., Meng Q., Wang C., Huo X., Liu Z., Wang C., Sun P., Sun H., Ma X. (2019). Catalpol Alleviates Adriamycin-Induced Nephropathy by Activating the Sirt1 Signalling Pathway In Vivo and In Vitro. Br. J. Pharmacol..

[116] Cong C., Yuan X., Hu Y., Chen W., Wang Y., Tao L. (2022). Catalpol Alleviates Ang Ii-Induced Renal Injury through Nf-Κb Pathway and Tgf-Β1/Smads Pathway. J. Cardiovasc. Pharmacol..

[117] Chen Y., Liu Q., Meng X., Zhao L., Zheng X., Feng W. (2024). Catalpol Ameliorates Fructose-Induced Renal Inflammation by Inhibiting Tlr4/Myd88 Signaling and Uric Acid Reabsorption. Eur. J. Pharmacol..

[118] Zaaba N.E., Al-Salam S., Beegam S., Elzaki O., Yasin J., Nemmar A. (2023). Catalpol Attenuates Oxidative Stress and Inflammation Via Mechanisms Involving Sirtuin-1 Activation and Nf-Κb Inhibition in Experimentally-Induced Chronic Kidney Disease. Nutrients.

[119] Zhang J., Zhao T., Wang C., Meng Q., Huo X., Wang C., Sun P., Sun H., Ma X., Wu J. (2021). Catalpol-Induced Ampk Activation Alleviates Cisplatin-Induced Nephrotoxicity through the Mitochondrial-Dependent Pathway without Compromising Its Anticancer Properties. Oxid. Med. Cell Longev..

[120] Chen J., Yang Y., Lv Z., Shu A., Du Q., Wang W., Chen Y., Xu H. (2020). Study on the Inhibitive Effect of Catalpol on Diabetic Nephropathy. Life Sci..

[121] Shu A., Du Q., Chen J., Gao Y., Zhu Y., Lv G., Lu J., Chen Y., Xu H. (2021). Catalpol Ameliorates Endothelial Dysfunction and Inflammation in Diabetic Nephropathy Via Suppression of Rage/Rhoa/Rock Signaling Pathway. Chem.-Biol. Interact..

[122] Zhang J., Wang C., Kang K., Liu H., Liu X., Jia X., Yu K. (2021). Loganin Attenuates Septic Acute Renal Injury with the Participation of Akt and Nrf2/Ho-1 Signaling Pathways. Drug Des. Devel Ther..

[123] Huang F., Wang X., Xiao G., Xiao J. (2022). Loganin Exerts a Protective Effect on Ischemia-Reperfusion-Induced Acute Kidney Injury by Regulating Jak2/Stat3 and Nrf2/Ho-1 Signaling Pathways. Drug Dev. Res..

[124] Kong X., Zhao Y., Wang X., Yu Y., Meng Y., Yan G., Yu M., Jiang L., Song W., Wang B. (2023). Loganin Reduces Diabetic Kidney Injury by Inhibiting the Activation of Nlrp3 Inflammasome-Mediated Pyroptosis. Chem.-Biol. Interact..

[125] Liu K., Xu H., Lv G., Liu B., Lee M.K.K., Lu C., Lv X., Wu Y. (2015). Loganin Attenuates Diabetic Nephropathy in C57bl/6j Mice with Diabetes Induced by Streptozotocin and Fed with Diets Containing High Level of Advanced Glycation End Products. Life Sci..

[126] Li X., Ma A., Liu K. (2019). Geniposide Alleviates Lipopolysaccharide-Caused Apoptosis of Murine Kidney Podocytes by Activating Ras/Raf/Mek/Erk-Mediated Cell Autophagy. Artif. Cells Nanomed. Biotechnol..

[127] Liu J., Zhao N., Shi G., Wang H. (2020). Geniposide Ameliorated Sepsis-Induced Acute Kidney Injury by Activating Pparγ. Aging.

[128] Liu X., Qian N., Zhu L., Fan L., Fu G., Ma M., Bao J., Cao C., Liang X. (2023). Geniposide Ameliorates Acute Kidney Injury Via Enhancing the Phagocytic Ability of Macrophages Towards Neutrophil Extracellular Traps. Eur. J. Pharmacol..

[129] Cheng W., Tan L., Yu S., Song J., Li Z., Peng X., Wei Q., He Z., Zhang W., Yang X. (2024). Geniposide Reduced Oxidative Stress-Induced Apoptosis in Hk-2 Cell through Pi3k/Akt3/Foxo1 by M6a Modification. Int. Immunopharmacol..

[130] Hu X., Zhang X., Jin G., Shi Z., Sun W., Chen F. (2017). Geniposide Reduces Development of Streptozotocin-Induced Diabetic Nephropathy Via Regulating Nuclear Factor-Kappa B Signaling Pathways Geniposide Reduces Development of Streptozotocin-Induced Diabetic Nephropathy Via Regulating Nuclear Factor-Kappa B Signaling Pathways. Fundam. Clin. Pharmacol..

[131] Dusabimana T., Park E.J., Je J., Jeong K., Yun S.P., Kim H.J., Kim H., Park S.W. (2021). Geniposide Improves Diabetic Nephropathy by Enhancing Ulk1-Mediated Autophagy and Reducing Oxidative Stress through Ampk Activation. Int. J. Mol. Sci..

[132] Peng J.H., Leng J., Tian H.J., Yang T., Fang Y., Feng Q., Zhao Y., Hu Y.Y. (2018). Geniposide and Chlorogenic Acid Combination Ameliorates Non-Alcoholic Steatohepatitis Involving the Protection on the Gut Barrier Function in Mouse Induced by High-Fat Diet. Front. Pharmacol..

[133] Gao X., Liu Y., Wang L., Sai N., Liu Y., Ni J. (2020). Morroniside Inhibits H_2_O_2_-Induced Podocyte Apoptosis by Down-Regulating Nox4 Expression Controlled by Autophagy in Vitro. Front. Pharmacol..

[134] Liu Y., Dai W., Ye S. (2019). The Olive Constituent Oleuropein Exerts Nephritic Protective Effects on Diabetic Nephropathy in Db/Db Mice. Arch. Physiol. Biochem..

[135] Tüfekci K.K., Tatar M. (2023). Oleuropein Mitigates Acrylamide-Induced Nephrotoxicity by Affecting Placental Growth Factor Immunoactivity in the Rat Kidney. Eurasian J. Med..

[136] Chen L., Cao G., Wang M., Feng Y.L., Chen D.Q., Vaziri N.D., Zhuang S., Zhao Y.Y. (2019). The Matrix Metalloproteinase-13 Inhibitor Poricoic Acid Zi Ameliorates Renal Fibrosis by Mitigating Epithelial-Mesenchymal Transition. Mol. Nutr. Food Res..

[137] Wang M., Chen D.Q., Chen L., Cao G., Zhao H., Liu D., Vaziri N.D., Guo Y., Zhao Y.Y. (2018). Novel Inhibitors of the Cellular Renin-Angiotensin System Components, Poricoic Acids, Target Smad3 Phosphorylation and Wnt/Β-Catenin Pathway against Renal Fibrosis. Br. J. Pharmacol..

[138] Chen D.Q., Wang Y.N., Vaziri N.D., Chen L., Hu H.H., Zhao Y.Y. (2020). Poricoic Acid a Activates Ampk to Attenuate Fibroblast Activation and Abnormal Extracellular Matrix Remodelling in Renal Fibrosis. Phytomedicine.

[139] Chen D.Q., Wu X.Q., Chen L., Hu H.H., Wang Y.N., Zhao Y.Y. (2020). Poricoic Acid a as a Modulator of Tph-1 Expression Inhibits Renal Fibrosis Via Modulating Protein Stability of Β-Catenin and Β-Catenin-Mediated Transcription. Ther. Adv. Chronic Dis..

[140] Chen D.Q., Feng Y.L., Chen L., Liu J.R., Wang M., Vaziri N.D., Zhao Y.Y. (2019). Poricoic Acid a Enhances Melatonin Inhibition of Aki-to-Ckd Transition by Regulating Gas6/Axlnfκb/Nrf2 Axis. Free Radic. Biol. Med..

[141] Chen D.Q., Chen L., Guo Y., Wu X.Q., Zhao T.T., Zhao H.L., Zhang H.J., Yan M.H., Zhang G.Q., Li P. (2023). Poricoic Acid a Suppresses Renal Fibroblast Activation and Interstitial Fibrosis in Uuo Rats Via Upregulating Sirt3 and Promoting Β-Catenin K49 Deacetylation. Acta Pharmacol. Sin..

[142] Li Q., Ming Y., Jia H., Wang G. (2021). Poricoic Acid a Suppresses Tgf-Β1-Induced Renal Fibrosis and Proliferation Via the Pdgf-C, Smad3 and Mapk Pathways. Exp. Ther. Med..

[143] Chung S., Yoon H.E., Kim S.J., Kim S.J., Koh E.S., Hong Y.A., Park C.W., Chang Y.S., Shin S.J. (2014). Oleanolic Acid Attenuates Renal Fibrosis in Mice with Unilateral Ureteral Obstruction Via Facilitating Nuclear Translocation of Nrf2. Nutr. Metab..

[144] Nataraju A., Saini D., Ramachandran S., Benshoff N., Liu W., Chapman W., Mohanakuma T.r. (2009). Oleanolic Acid, a Plant Triterpenoid, Significantly Improves Survival and Function of Islet Allograft. Transplantation.

[145] Alqrad M.A.I., El-Agamy D.S., Ibrahim S.R.M., Sirwi A., Abdallah H.M., Abdel-Sattar E., El-Halawany A.M., Elsaed W.M., Mohamed G.A. (2023). Sirt1/Nrf2/Nf-Κb Signaling Mediates Anti-Inflammatory and Anti-Apoptotic Activities of Oleanolic Acid in a Mouse Model of Acute Hepatorenal Damage. Medicina.

[146] Liu Y., Zheng J.Y., Wei Z.T., Liu S.K., Sun J.L., Mao Y.H., Xu Y.D., Yang Y. (2022). Therapeutic Effect and Mechanism of Combination Therapy with Ursolic Acid and Insulin on Diabetic Nephropathy in a Type I Diabetic Rat Model. Front. Pharmacol..

[147] Wang K., Xu X., Shan Q., Ding R., Lyu Q., Huang L., Chen X., Han X., Yang Q., Sang X. (2022). Integrated Gut Microbiota and Serum Metabolomics Reveal the Protective Effect of Oleanolic Acid on Liver and Kidney-Injured Rats Induced by Euphorbia Pekinensis. Phytother. Res..

[148] Zhang J., Luan Z.L., Huo X.K., Zhang M., Morisseau C., Sun C.P., Hammock B.D., Ma X.C. (2023). Direct Targeting of Seh with Alisol B Alleviated the Apoptosis, Inflammation, and Oxidative Stress in Cisplatin-Induced Acute Kidney Injury. Int. J. Biol. Sci..

[149] Luan Z.L., Ming W.H., Sun X.W., Zhang C., Zhou Y., Zheng F., Yang Y.L., Guan Y.F., Zhang X.Y. (2021). A Naturally Occurring Fxr Agonist, Alisol B 23-Acetate, Protects against Renal Ischemia-Reperfusion Injury. Am. J. Physiol. Renal Physiol..

[150] Chen H., Wang M.C., Chen Y.Y., Chen L., Wang Y.N., Vaziri N.D., Miao H., Zhao Y.Y. (2020). Alisol B 23-Acetate Attenuates Ckd Progression by Regulating the Renin-Angiotensin System and Gut-Kidney Axis. Ther. Adv. Chronic Dis..

[151] Wang L., Hao X., Li X., Li Q., Fang X. (2024). Effects of Ginsenoside Rh2 on Cisplatin-Induced Nephrotoxicity in Renal Tubular Epithelial Cells by Inhibiting Endoplasmic Reticulum Stress. J. Biochem. Mol. Toxicol..

[152] Guo J., Chen L., Ma M. (2024). Ginsenoside Rg1 Suppresses Ferroptosis of Renal Tubular Epithelial Cells in Sepsis-Induced Acute Kidney Injury Via the Fsp1-Coq(10)-Nad(P)H Pathway. Curr. Med. Chem..

[153] Zhao H., Ding R., Han J. (2024). Ginsenoside Rh4 Facilitates the Sensitivity of Renal Cell Carcinoma to Ferroptosis Via the Nrf2 Pathway. Arch. Esp. Urol..

[154] Hwang H.J., Hong S.H., Moon H.S., Yoon Y.E., Park S.Y. (2022). Ginsenoside Rh2 Sensitizes the Anti-Cancer Effects of Sunitinib by Inducing Cell Cycle Arrest in Renal Cell Carcinoma. Sci. Rep..

[155] Chen Y., Peng Y., Li P., Jiang Y., Song D. (2024). Ginsenoside Rg3 Induces Mesangial Cells Proliferation and Attenuates Apoptosis by Mir-216a-5p/Mapk Pathway in Diabetic Kidney Disease. Aging.

[156] Sui Z., Sui D., Li M., Yu Q., Li H., Jiang Y. (2023). Ginsenoside Rg3 Has Effects Comparable to Those of Ginsenoside Re on Diabetic Kidney Disease Prevention in Db/Db Mice by Regulating Inflammation, Fibrosis and Pparγ. Mol. Med. Rep..

[157] Liu Y., Mou L., Yi Z., Lin Q., Banu K., Wei C., Yu X. (2024). Integrative Informatics Analysis Identifies That Ginsenoside Re Improves Renal Fibrosis through Regulation of Autophagy. J. Nat. Med..

[158] Ji P., Shi Q., Liu Y., Han M., Su Y., Sun R., Zhou H., Li W., Li W. (2023). Ginsenoside Rg1 Treatment Alleviates Renal Fibrosis by Inhibiting the Nox4-Mapk Pathway in T2dm Mice. Ren. Fail..

[159] Su L., Liu L., Jia Y., Lei L., Liu J., Zhu S., Zhou H., Chen R., Lu H.A.J., Yang B. (2017). Ganoderma Triterpenes Retard Renal Cyst Development by Downregulating Ras/Mapk Signaling and Promoting Cell Differentiation. Kidney Int..

[160] Geng X.Q., Ma A., He J.Z., Wang L., Jia Y.L., Shao G.Y., Li M., Zhou H., Lin S.Q., Ran J.H. (2020). Ganoderic Acid Hinders Renal Fibrosis Via Suppressing the Tgf-Β/Smad and Mapk Signaling Pathways. Acta Pharmacol. Sin..

[161] Shao G., He J., Meng J., Ma A., Geng X., Zhang S., Qiu Z., Lin D., Li M., Zhou H. (2021). Ganoderic Acids Prevent Renal Ischemia Reperfusion Injury by Inhibiting Inflammation and Apoptosis. Int. J. Mol. Sci..

[162] Cai L., Horowitz M., Islam M.S. (2023). Potential Therapeutic Targets for the Prevention of Diabetic Nephropathy: Glycyrrhetinic Acid. World J. Diabetes.

[163] Hou S., Zhang T., Li Y., Guo F., Jin X. (2017). Glycyrrhizic Acid Prevents Diabetic Nephropathy by Activating Ampk/Sirt1/Pgc-1α Signaling in Db/Db Mice. J. Diabetes Res..

[164] Cao R., Li Y., Hu X., Qiu Y., Li S., Xie Y., Xu C., Lu C., Chen G., Yang J. (2023). Glycyrrhizic Acid Improves Tacrolimus-Induced Renal Injury by Regulating Autophagy. Faseb J..

[165] Oh H., Choi A., Seo N., Lim J.S., You J.S., Chung Y.E. (2021). Protective Effect of Glycyrrhizin, a Direct Hmgb1 Inhibitor, on Post-Contrast Acute Kidney Injury. Sci. Rep..

[166] Li Z., Yan M., Cao L., Fang P., Guo Z., Hou Z., Zhang B. (2017). Glycyrrhetinic Acid Accelerates the Clearance of Triptolide through P-Gp in Vitro. Phytother. Res..

[167] Jiang Y., Cai C., Zhang P., Luo Y., Guo J., Li J., Rong R., Zhang Y., Zhu T. (2022). Transcriptional Profile Changes after Treatment of Ischemia Reperfusion Injury-Induced Kidney Fibrosis with 18β-Glycyrrhetinic Acid. Ren. Fail..

[168] Zhu Z., Li J., Song Z., Li T., Li Z., Gong X. (2024). Tetramethylpyrazine Attenuates Renal Tubular Epithelial Cell Ferroptosis in Contrast-Induced Nephropathy by Inhibiting Transferrin Receptor and Intracellular Reactive Oxygen Species. Clin. Sci..

[169] Gong X., Duan Y., Zheng J., Ye Z., Hei T.K. (2019). Tetramethylpyrazine Prevents Contrast-Induced Nephropathy Via Modulating Tubular Cell Mitophagy and Suppressing Mitochondrial Fragmentation, Ccl2/Ccr2-Mediated Inflammation, and Intestinal Injury. Oxid. Med. Cell Longev..

[170] Sun W., Li A., Wang Z., Sun X., Dong M., Qi F., Wang L., Zhang Y., Du P. (2020). Tetramethylpyrazine Alleviates Acute Kidney Injury by Inhibiting Nlrp3/Hif-1α and Apoptosis. Mol. Med. Rep..

[171] Rai U., Kosuru R., Prakash S., Tiwari V., Singh S. (2019). Tetramethylpyrazine Alleviates Diabetic Nephropathy through the Activation of Akt Signalling Pathway in Rats. Eur. J. Pharmacol..

[172] Jing M., Cen Y., Gao F., Wang T., Jiang J., Jian Q., Wu L., Guo B., Luo F., Zhang G. (2021). Nephroprotective Effects of Tetramethylpyrazine Nitrone Tbn in Diabetic Kidney Disease. Front. Pharmacol..

[173] Hu J., Gu W., Ma N., Fan X., Ci X. (2022). Leonurine Alleviates Ferroptosis in Cisplatin-Induced Acute Kidney Injury by Activating the Nrf2 Signalling Pathway. Br. J. Pharmacol..

[174] Yin X., Gao Q., Li C., Yang Q., Dong H., Li Z. (2024). Leonurine Alleviates Vancomycin Nephrotoxicity Via Activating Pparγ and Inhibiting the Tlr4/Nf-Κb/Tnf-A Pathway. Int. Immunopharmacol..

[175] Cheng H., Bo Y., Shen W., Tan J., Jia Z., Xu C., Li F. (2015). Leonurine Ameliorates Kidney Fibrosis Via Suppressing Tgf-Β and Nf-Κb Signaling Pathway in Uuo Mice. Int. Immunopharmacol..

[176] Xu D., Chen M., Ren X., Ren X., Wu Y. (2014). Leonurine Ameliorates Lps-Induced Acute Kidney Injury Via Suppressing Ros-Mediated Nf-Κb Signaling Pathway. Fitoterapia.

[177] Cheng R., Wang X., Huang L., Lu Z., Wu A., Guo S., Li C., Mao W., Xie Y., Xu P. (2024). Novel Insights into the Protective Effects of Leonurine against Acute Kidney Injury: Inhibition of Er Stress-Associated Ferroptosis Via Regulating Atf4/Chop/Acsl4 Pathway. Chem. Biol. Interact..

[178] Hassanein E.H.M., Shalkami A.-G.S., Khalaf M.M., Mohamed W.R., Hemeida R.A.M. (2019). The Impact of Keap1/Nrf2, P_38_Mapk/Nf-Κb and Bax/Bcl2/Caspase-3 Signaling Pathways in the Protective Effects of Berberine against Methotrexate-Induced Nephrotoxicity. Biomed. Pharmacother..

[179] Fouad G.I., Ahmed K.A. (2021). The Protective Impact of Berberine against Doxorubicin-Induced Nephrotoxicity in Rats. Tissue Cell.

[180] Malaviya A.N. (2016). Landmark Papers on the Discovery of Methotrexate for the Treatment of Rheumatoid Arthritis and Other Systemic Inflammatory Rheumatic Diseases: A Fascinating Story. Int. J. Rheum. Dis..

[181] Domitrovic R., Cvijanovic O., Pernjak-Pugel E., Skoda M., Mikelic L., Crncevic-Orlic Z. (2013). Berberine Exerts Nephroprotective Effect against Cisplatin-Induced Kidney Damage through Inhibition of Oxidative/Nitrosative Stress, Inflammation, Autophagy and Apoptosis. Food Chem. Toxicol..

[182] Adil M., Kandhare A.D., Dalvi G., Ghosh P., Venkata S., Raygude K.S., Bodhankar S.L. (2016). Ameliorative Effect of Berberine against Gentamicin-Induced Nephrotoxicity in Rats Via Attenuation of Oxidative Stress, Inflammation, Apoptosis and Mitochondrial Dysfunction. Ren. Fail..

[183] Kumas M., Esrefoglu M., Karatas E., Duymac N., Kanbay S., Ergun I.S., Uyuklu M., Kocyigit A. (2019). Investigation of Dose-Dependent Effects of Berberine against Renal Ischemia/Reperfusion Injury in Experimental Diabetic Rats. Nefrologia.

[184] Lu J., Yi Y., Pan R., Zhang C., Han H., Chen J., Liu W. (2018). Berberine Protects Hk-2 Cells from Hypoxia/Reoxygenation Induced Apoptosis Via Inhibiting Sphk1 Expression. J. Nat. Med..

[185] Visnagri A., Kandhare A.D., Bodhankar S.L. (2015). Renoprotective Effect of Berberine Via Intonation on Apoptosis and Mitochondrial-Dependent Pathway in Renal Ischemia Reperfusion-Induced Mutilation. Ren. Fail..

[186] Zhang X., He H., Liang D., Jiang Y., Liang W., Chi Z.-H., Ma J. (2016). Protective Effects of Berberine on Renal Injury in Streptozotocin (Stz)-Induced Diabetic Mice. Int. J. Mol. Sci..

[187] Yang G., Zhao Z., Zhang X., Wu A., Huang Y., Miao Y., Yang M. (2017). Effect of Berberine on the Renal Tubular Epithelial-to- Mesenchymal Transition by Inhibition of the Notch/Snail Pathway in Diabetic Nephropathy Model Kkay Mice. Drug Des. Dev. Ther..

[188] Zhang X., Guan T., Yang B., Chi Z., Wan Q., Gu H.F. (2019). Protective Effect of Berberine on High Glucose and Hypoxia-Induced Apoptosis Via the Modulation of Hif-1α in Renal Tubular Epithelial Cells. Am. J. Transl. Res..

[189] Wang F.-M., Yang Y.-J., Ma L.-L., Tian X.-J., He Y.-Q. (2014). Berberine Ameliorates Renal Interstitial Fibrosis Induced by Unilateral Ureteral Obstruction in Rats. Nephrology.

[190] Pan L., Yu H., Fu J., Hu J., Xu H., Zhang Z., Bu M., Yang X., Zhang H., Lu J. (2023). Berberine Ameliorates Chronic Kidney Disease through Inhibiting the Production of Gut-Derived Uremic Toxins in the Gut Microbiota. Acta Pharm. Sin. B.

[191] Peerapen P., Thongboonkerd V. (2020). Protective Roles of Trigonelline against Oxalate-Induced Epithelial-to-Mesenchymal Transition in Renal Tubular Epithelial Cells: An in Vitro Study. Food Chem. Toxicol..

[192] Peerapen P., Boonmark W., Putpeerawit P., Sassanarakkit S., Thongboonkerd V. (2023). Proteomic and Computational Analyses Followed by Functional Validation of Protective Effects of Trigonelline against Calcium Oxalate-Induced Renal Cell Deteriorations. Comput. Struct. Biotechnol. J..

[193] Sheweita S.A., ElHady S.A., Hammoda H.M. (2020). Trigonella Stellata Reduced the Deleterious Effects of Diabetes Mellitus through Alleviation of Oxidative Stress, Antioxidant- and Drug-Metabolizing Enzymes Activities. J. Ethnopharmacol..

[194] Chen C., Ma J., Miao C.S., Zhang H., Zhang M., Cao X., Shi Y. (2021). Trigonelline Induces Autophagy to Protect Mesangial Cells in Response to High. Glucose Via Activating the Mir-5189-5p-Ampk Pathway. Phytomedicine.

[195] Gong M., Guo Y., Dong H., Wu W., Wu F., Lu F. (2023). Trigonelline Inhibits Tubular Epithelial-Mesenchymal Transformation in Diabetic Kidney Disease Via Targeting Smad7. Biomed. Pharmacother..

[196] Peerapen P., Boonmark W., Thongboonkerd V. (2022). Trigonelline Prevents Kidney Stone Formation Processes by Inhibiting Calcium Oxalate Crystallization, Growth and Crystal-Cell Adhesion, and Downregulating Crystal Receptors. Biomed. Pharmacother..

[197] Zhou L., Wu K., Gao Y., Qiao R., Tang N., Dong D., Li X.Q., Nong Q., Luo D.Q., Xiao Q. (2023). Piperlonguminine Attenuates Renal Fibrosis by Inhibiting Trpc6. J. Ethnopharmacol..

[198] Yuan L., Yang J., Li Y., Yuan L., Liu F., Yuan Y., Tang X. (2022). Matrine Alleviates Cisplatin-Induced Acute Kidney Injury by Inhibiting Mitochondrial Dysfunction and Inflammation Via Sirt3/Opa1 Pathway. J. Cell Mol. Med..

[199] Kang S., Chen T., Hao Z., Yang X., Wang M., Zhang Z., Hao S., Lang F., Hao H. (2022). Oxymatrine Alleviates Gentamicin-Induced Renal Injury in Rats. Molecules.

[200] Jiang G., Liu X., Wang M., Chen H., Chen Z., Qiu T. (2015). Oxymatrine Ameliorates Renal Ischemia-Reperfusion Injury from Oxidative Stress through Nrf2/Ho-1 Pathway. Acta Cir. Bras..

[201] Yao M., Lian D., Wu M., Zhou Y., Fang Y., Zhang S., Zhang W., Yang Y., Li R., Chen H. (2023). Isoliensinine Attenuates Renal Fibrosis and Inhibits Tgf-Β1/Smad2/3 Signaling Pathway in Spontaneously Hypertensive Rats. Drug Des. Devel Ther..

[202] Zhang W., Chen H., Xu Z., Zhang X., Tan X., He N., Shen J., Dong J. (2023). Liensinine Pretreatment Reduces Inflammation, Oxidative Stress, Apoptosis, and Autophagy to Alleviate Sepsis Acute Kidney Injury. Int. Immunopharmacol..

[203] Xiong Y., Zhong J., Chen W., Li X., Liu H., Li Y., Xiong W., Li H. (2024). Neferine Alleviates Acute Kidney Injury by Regulating the Ppar-A/Nf-Κb Pathway. Clin. Exp. Nephrol..

[204] Li H., Chen W., Chen Y., Zhou Q., Xiao P., Tang R., Xue J. (2019). Neferine Attenuates Acute Kidney Injury by Inhibiting Nf-Κb Signaling and Upregulating Klotho Expression. Front. Pharmacol..

[205] Hongmei H., Maojun Y., Ting L.I., Dandan W., Ying L.I., Xiaochi T., Lu Y., Shi G.U., Yong X.U. (2024). Neferine Inhibits the Progression of Diabetic Nephropathy by Modulating the Mir-17-5p/Nuclear Factor E2-Related Factor 2 Axis. J. Tradit. Chin. Med..

[206] Li H., Ge H., Song X., Tan X., Xiong Q., Gong Y., Zhang L., He Y., Zhang W., Zhu P. (2023). Neferine Mitigates Cisplatin-Induced Acute Kidney Injury in Mice by Regulating Autophagy and Apoptosis. Clin. Exp. Nephrol..

[207] Li H., Tang Y., Wen L., Kong X., Chen X., Liu P., Zhou Z., Chen W., Xiao C., Xiao P. (2017). Neferine Reduces Cisplatin-Induced Nephrotoxicity by Enhancing Autophagy Via the Ampk/Mtor Signaling Pathway. Biochem. Biophys. Res. Commun..

[208] Yin W., Wang J.H., Liang Y.M., Liu K.H., Chen Y., Chen Y. (2024). Neferine Targeted the Nlrc5/Nlrp3 Pathway to Inhibit M1-Type Polarization and Pyroptosis of Macrophages to Improve Hyperuricemic Nephropathy. Curr. Mol. Med..

[209] Wang M.X., Zhao X.J., Chen T.Y., Liu Y.L., Jiao R.Q., Zhang J.H., Ma C.H., Liu J.H., Pan Y., Kong L.D. (2016). Nuciferine Alleviates Renal Injury by Inhibiting Inflammatory Responses in Fructose-Fed Rats. J. Agric. Food Chem..

[210] Li D., Liu B., Fan Y., Liu M., Han B., Meng Y., Xu X., Song Z., Liu X., Hao Q. (2021). Nuciferine Protects against Folic Acid-Induced Acute Kidney Injury by Inhibiting Ferroptosis. Br. J. Pharmacol..

[211] Zhai J., Chen Z., Zhu Q., Guo Z., Sun X., Jiang L., Li J., Wang N., Yao X., Zhang C. (2024). Curcumin Inhibits Pat-Induced Renal Ferroptosis Via the P62/Keap1/Nrf2 Signalling Pathway. Toxicology.

[212] Yang H., Zhang H., Tian L., Guo P., Liu S., Chen H., Sun L. (2024). Curcumin Attenuates Lupus Nephritis by Inhibiting Neutrophil Migration Via Pi3k/Akt/Nf-Κb Signalling Pathway. Lupus Sci. Med..

[213] Ghasemzadeh Rahbardar M., Hosseinzadeh H. (2024). The Ameliorative Effect of Turmeric (*Curcuma longa* Linn) Extract and Its Major Constituent, Curcumin, and Its Analogs on Ethanol Toxicity. Phytother. Res..

[214] Zhang H., Dong Q.Q., Shu H.P., Tu Y.C., Liao Q.Q., Yao L.J. (2023). Curcumin Ameliorates Focal Segmental Glomerulosclerosis by Inhibiting Apoptosis and Oxidative Stress in Podocytes. Arch. Biochem. Biophys..

[215] Altamimi J.Z., AlFaris N.A., Al-Farga A.M., Alshammari G.M., BinMowyna M.N., Yahya M.A. (2021). Curcumin Reverses Diabetic Nephropathy in Streptozotocin-Induced Diabetes in Rats by Inhibition of Pkcβ/P(66)Shc Axis and Activation of Foxo-3a. J. Nutr. Biochem..

[216] Feng L., Lin Z., Tang Z., Zhu L., Xu S., Tan X., Wang X., Mai J., Tan Q. (2024). Emodin Improves Renal Fibrosis in Chronic Kidney Disease by Regulating Mitochondrial Homeostasis through the Mediation of Peroxisome Proliferator-Activated Receptor-Gamma Coactivator-1 Alpha (Pgc-1α). Eur. J. Histochem..

[217] Dong X., Wen R., Xiong Y., Jia X., Zhang X., Li X., Zhang L., Li Z., Zhang S., Yu Y. (2024). Emodin Alleviates Crs4-Induced Mitochondrial Damage Via Activation of the Pgc1α Signaling. Phytother. Res..

[218] Wang L., Wang X., Li G., Zhou S., Wang R., Long Q., Wang M., Li L., Huang H., Ba Y. (2023). Emodin Ameliorates Renal Injury and Fibrosis Via Regulating the Mir-490-3p/Hmga2 Axis. Front. Pharmacol..

[219] Yang F., Deng L., Li J., Chen M., Liu Y., Hu Y., Zhong W. (2020). Emodin Retarded Renal Fibrosis through Regulating Hgf and Tgfβ-Smad Signaling Pathway. Drug Des. Devel Ther..

[220] Tian N., Gao Y., Wang X., Wu X., Zou D., Zhu Z., Han Z., Wang T., Shi Y. (2018). Emodin Mitigates Podocytes Apoptosis Induced by Endoplasmic Reticulum Stress through the Inhibition of the Perk Pathway in Diabetic Nephropathy. Drug Des. Devel Ther..

[221] Kuang B.C., Wang Z.H., Hou S.H., Zhang J., Wang M.Q., Zhang J.S., Sun K.L., Ni H.Q., Gong N.Q. (2023). Methyl Eugenol Protects the Kidney from Oxidative Damage in Mice by Blocking the Nrf2 Nuclear Export Signal through Activation of the Ampk/Gsk3β Axis. Acta Pharmacol. Sin..

[222] Fathy M., Abdel-Latif R., Abdelgwad Y.M., Othman O.A., Abdel-Razik A.H., Dandekar T., Othman E.M. (2022). Nephroprotective Potential of Eugenol in a Rat Experimental Model of Chronic Kidney Injury; Targeting Nox, Tgf-Β, and Akt Signaling. Life Sci..

[223] Oikawa D., Yamashita S., Takahashi S., Waki T., Kikuchi K., Abe T., Katayama T., Nakayama T. (2022). (+)-Sesamin, a Sesame Lignan, Is a Potent Inhibitor of Gut Bacterial Tryptophan Indole-Lyase That Is a Key Enzyme in Chronic Kidney Disease Pathogenesis. Biochem. Biophys. Res. Commun..

[224] Altyar A.E., Albadrani G.M., Farouk S.M., Alamoudi M.K., Sayed A.A., Mohammedsaleh Z.M., Al-Ghadi M.Q., Saleem R.M., Sakr H.I., Abdel-Daim M.M. (2024). The Antioxidant, Anti-Inflammatory, and Anti-Apoptotic Effects of Sesamin against Cisplatin-Induced Renal and Testicular Toxicity in Rats. Ren. Fail..

[225] Rousta A.M., Mirahmadi S.M., Shahmohammadi A., Nourabadi D., Khajevand-Khazaei M.R., Baluchnejadmojarad T., Roghani M. (2018). Protective Effect of Sesamin in Lipopolysaccharide-Induced Mouse Model of Acute Kidney Injury Via Attenuation of Oxidative Stress, Inflammation, and Apoptosis. Immunopharmacol. Immunotoxicol..

[226] Zhang R., Yu Y., Deng J., Zhang C., Zhang J., Cheng Y., Luo X., Han B., Yang H. (2016). Sesamin Ameliorates High-Fat Diet-Induced Dyslipidemia and Kidney Injury by Reducing Oxidative Stress. Nutrients.

[227] Zhang N., Zhou J., Zhao L., Zhao Z., Wang S., Zhang L., Zhou F. (2023). Ferulic Acid Supplementation Alleviates Hyperuricemia in High-Fructose/Fat Diet-Fed Rats Via Promoting Uric Acid Excretion and Mediating the Gut Microbiota. Food Funct..

[228] Zhou X., Zhang B., Zhao X., Lin Y., Zhuang Y., Guo J., Wang S. (2022). Chlorogenic Acid Prevents Hyperuricemia Nephropathy Via Regulating Tmao-Related Gut Microbes and Inhibiting the Pi3k/Akt/Mtor Pathway. J. Agric. Food Chem..

[229] Zhou X., Zhang B., Zhao X., Lin Y., Wang J., Wang X., Hu N., Wang S. (2021). Chlorogenic Acid Supplementation Ameliorates Hyperuricemia, Relieves Renal Inflammation, and Modulates Intestinal Homeostasis. Food Funct..

[230] Qu S., Dai C., Hao Z., Tang Q., Wang H., Wang J., Zhao H. (2020). Chlorogenic Acid Prevents Vancomycin-Induced Nephrotoxicity without Compromising Vancomycin Antibacterial Properties. Phytother. Res..

[231] Li H.-Y., Yi Y.-L., Guo S., Zhang F., Yan H., Zhan Z.-L., Zhu Y., Duan J.-A. (2022). Isolation, Structural Characterization and Bioactivities of Polysaccharides from Laminaria Japonica: A Review. Food Chem..

[232] Lu J., Yao J., Pu J., Wang D., Liu J., Zhang Y., Zha L. (2023). Transcriptome Analysis of Three Medicinal Plants of the Genus Polygonatum: Identification of Genes Involved in Polysaccharide and Steroidal Saponins Biosynthesis. Front. Plant Sci..

[233] Ge J., Liu Z., Zhong Z., Wang L., Zhuo X., Li J., Jiang X., Ye X.-Y., Xie T., Bai R. (2022). Natural Terpenoids with Anti-Inflammatory Activities: Potential Leads for Anti-Inflammatory Drug Discovery. Bioorganic Chem..

[234] Galappaththi M.C.A., Patabendige N.M., Premarathne B.M., Hapuarachchi K.K., Tibpromma S., Dai D.-Q., Suwannarach N., Rapior S., Karunarathna S.C. (2022). A Review of Ganoderma Triterpenoids and Their Bioactivities. Biomolecules.

[235] Lu D., Yang Y., Du Y., Zhang L., Yang Y., Tibenda J.J., Nan Y., Yuan L. (2023). The Potential of Glycyrrhiza from “Medicine Food Homology” in the Fight against Digestive System Tumors. Molecules.

[236] Wu Y., Wang X., Yang L., Kang S., Yan G., Han Y., Fang H., Sun H. (2023). Potential of Alisols as Cancer Therapeutic Agents: Investigating Molecular Mechanisms, Pharmacokinetics and Metabolism. Biomed. Pharmacother..

[237] Lee C.H., Kim J.H. (2014). A Review on the Medicinal Potentials of Ginseng and Ginsenosides on Cardiovascular Diseases. J. Ginseng Res..

[238] Yokozawa T., Liu Z.W., Dong E. (1998). A Study of Ginsenoside-Rd in a Renal Ischemia-Reperfusion Model. Nephron.

[239] Kouda R., Yakushiji F. (2020). Recent Advances in Iridoid Chemistry: Biosynthesis and Chemical Synthesis. Chem.—Asian J..

[240] Danielewski M., Matuszewska A., Nowak B., Kucharska A.Z., Sozański T. (2020). The Effects of Natural Iridoids and Anthocyanins on Selected Parameters of Liver and Cardiovascular System Functions. Oxid. Med. Cell Longev..

[241] Bridi R., von Poser G.L., de Carvalho Meirelles G. (2023). Iridoids as a Potential Hepatoprotective Class: A Review. Mini Rev. Med. Chem..

[242] Zhou T.Y., Tian N., Li L., Yu R. (2024). Iridoids Modulate Inflammation in Diabetic Kidney Disease: A Review. J. Integr. Med..

[243] Kou Y., Li Z., Yang T., Shen X., Wang X., Li H., Zhou K., Li L., Xia Z., Zheng X. (2022). Therapeutic Potential of Plant Iridoids in Depression: A Review. Pharm. Biol..

[244] Zhang F., Yan Y., Xu J.K., Zhang L.M., Li L., Chen X., Li D.X., Peng Y., Yang H., Li L.Z. (2024). Simultaneous Determination of Thirteen Iridoid Glycosides in Crude and Processed Fructus Corni from Different Areas by Uplc-Ms/Ms Method. J. Chromatogr. Sci..

[245] Cheng C., Li Z., Zhao X., Liao C., Quan J., Bode A.M., Cao Y., Luo X. (2020). Natural Alkaloid and Polyphenol Compounds Targeting Lipid Metabolism: Treatment Implications in Metabolic Diseases. Eur. J. Pharmacol..

[246] Li J., Gong X. (2022). Tetramethylpyrazine: An Active Ingredient of Chinese Herbal Medicine W Ith Therapeutic Potential in Acute Kidney Injury and Renal Fibrosis. Front. Pharmacol..

[247] Wu D., Wen W., Qi C.L., Zhao R.X., Lü J.H., Zhong C.Y., Chen Y.Y. (2012). Ameliorative Effect of Berberine on Renal Damage in Rats with Diabetes Induced by High-Fat Diet and Streptozotocin. Phytomedicine.

[248] Tang L., Lv F., Liu S., Zhang S. (2011). Effect of Berberine on Expression of Transforming Growth Factor-Beta1 and Type Iv Collagen Proteins in Mesangial Cells of Diabetic Rats with Nephropathy. Zhongguo Zhong Yao Za Zhi = Zhongguo Zhongyao Zazhi = China J. Chin. Mater. Medica.

[249] Chen Q., Li D., Wu F., He X., Zhou Y., Sun C., Wang H., Liu Y. (2023). Berberine Regulates the Metabolism of Uric Acid and Modulates Intestinal Flora in Hyperuricemia Rats Model. Comb. Chem. High. Throughput Screen..

[250] Shan B., Wu M., Chen T., Tang W., Li P., Chen J. (2022). Berberine Attenuates Hyperuricemia by Regulating Urate Transporters and Gut Microbiota. Am. J. Chin. Med..

[251] Pan L., Han P., Ma S., Peng R., Wang C., Kong W., Cong L., Fu J., Zhang Z., Yu H. (2020). Abnormal Metabolism of Gut Microbiota Reveals the Possible Molecular Mechanism of Nephropathy Induced by Hyperuricemia. Acta Pharm. Sin. B.

[252] Wang T., Zhang J., Wei H., Wang X., Xie M., Jiang Y., Zhou J. (2023). Matrine-Induced Nephrotoxicity Via Gsk-3β/Nrf2-Mediated Mitochondria-Dependent Apoptosis. Chem. Biol. Interact..

[253] Wang X., Lin Z., Li T., Zhu W., Huang H., Hu J., Zhou J. (2024). Sodium Selenite Prevents Matrine-Induced Nephrotoxicity by Suppressing Ferroptosis Via the Gsh-Gpx4 Antioxidant System. Biol. Trace Element Res..

[254] Wang H.-W., Shi L., Xu Y.-P., Qin X.-Y., Wang Q.-Z. (2016). Oxymatrine Inhibits Renal Fibrosis of Obstructive Nephropathy by Downregulating the Tgf-Β1-Smad3 Pathway. Ren. Fail..

[255] Zhu Z., Chen R., Zhang L., Zhu Z. (2024). Simple Phenylpropanoids: Recent Advances in Biological Activities, Biosynthetic Pathways, and Microbial Production. Nat. Prod. Rep..

[256] Zhu X., Quan Y.-Y., Yin Z.-J., Li M., Wang T., Zheng L.-Y., Feng S.-Q., Zhao J.-N., Li L. (2023). Sources, Morphology, Phytochemistry, Pharmacology of Curcumae Longae Rhizoma, Curcumae Radix, and Curcumae Rhizoma: A Review of the Literature. Front. Pharmacol..

[257] Pivari F., Mingione A., Piazzini G., Ceccarani C., Ottaviano E., Brasacchio C., Cas M.D., Vischi M., Cozzolino M.G., Fogagnolo P. (2022). Curcumin Supplementation (Meriva^®^) Modulates Inflammation, Lipid Peroxidation and Gut Microbiota Composition in Chronic Kidney Disease. Nutrients.

[258] Xu X., Wang H., Guo D., Man X., Liu J., Li J., Luo C., Zhang M., Zhen L., Liu X. (2021). Curcumin Modulates Gut Microbiota and Improves Renal Function in Rats with Uric Acid Nephropathy. Ren. Fail..

[259] Mohtashami L., Amiri M.S., Ayati Z., Ramezani M., Jamialahmadi T., Emami S.A., Sahebkar A. (2021). Ethnobotanical Uses, Phytochemistry and Pharmacology of Different Rheum Species (Polygonaceae): A Review. Adv. Exp. Med. Biol..

[260] Zeng Y.Q., Dai Z., Lu F., Lu Z., Liu X., Chen C., Qu P., Li D., Hua Z., Qu Y. (2016). Emodin Via Colonic Irrigation Modulates Gut Microbiota and Reduces Uremic Toxins in Rats with Chronic Kidney Disease. Oncotarget.

[261] Tucker P.S., Scanlan A.T., Dalbo V.J. (2015). Chronic Kidney Disease Influences Multiple Systems: Describing the Relationship between Oxidative Stress, Inflammation, Kidney Damage, and Concomitant Disease. Oxidative Med. Cell. Longev..

[262] Tain Y.-L., Hsu C.-N. (2023). Perinatal Oxidative Stress and Kidney Health: Bridging the Gap between Animal Models and Clinical Reality. Antioxidants.

[263] Mapuskar K.A., Pulliam C.F., Zepeda-Orozco D., Griffin B.R., Furqan M., Spitz D.R., Allen B.G. (2023). Redox Regulation of Nrf2 in Cisplatin-Induced Kidney Injury. Antioxidants.

[264] Honda T., Hirakawa Y., Nangaku M. (2019). The Role of Oxidative Stress and Hypoxia in Renal Disease. Kidney Res. Clin. Pract..

[265] Allameh H., Fatemi I., Malayeri A.R., Nesari A., Mehrzadi S., Goudarzi M. (2020). Pretreatment with Berberine Protects against Cisplatin-Induced Renal Injury in Male Wistar Rats. Naunyn-Schmiedebergs Arch. Pharmacol..

[266] Verma V.K., Malik S., Mutneja E., Sahu A.K., Rupashi K., Dinda A.K., Arya D.S., Bhatia J. (2020). Mechanism Involved in Fortification by Berberine in Cddp-Induced Nephrotoxicity. Curr. Mol. Pharmacol..

[267] El-Horany H.E.-S., Gaballah H.H., Helal D.S. (2020). Berberine Ameliorates Renal Injury in a Rat Model of D-Galactose-Induced Aging through a Pten/Akt-Dependent Mechanism. Arch. Physiol. Biochem..

[268] Hasanein P., Riahi H. (2018). Preventive Use of Berberine in Inhibition of Lead-Induced Renal Injury in Rats. Environ. Sci. Pollut. Res..

[269] Basile D.P., Anderson M.D., Sutton T.A. (2012). Pathophysiology of Acute Kidney Injury. Compr. Physiol..

[270] Fu Y., Xiang Y., Li H., Chen A., Dong Z. (2022). Inflammation in Kidney Repair: Mechanism and Therapeutic Potential. Pharmacol. Ther..

[271] Mosquera-Sulbaran J.A., Pedreañez A., Vargas R., Hernandez-Fonseca J.P. (2024). Apoptosis in Post-Streptococcal Glomerulonephritis and Mechanisms for Failed of Inflammation Resolution. Pediatr. Nephrol..

[272] Zhang H., Deng Z., Wang Y. (2023). Molecular Insight in Intrarenal Inflammation Affecting Four Main Types of Cells in Nephrons in Iga Nephropathy. Front. Med..

[273] Vallés P.G., Lorenzo A.F.G., Garcia R.D., Cacciamani V., Benardon M.E., Costantino V.V. (2023). Toll-Like Receptor 4 in Acute Kidney Injury. Int. J. Mol. Sci..

[274] Yeh T.H., Tu K.C., Wang H.Y., Chen J.Y. (2024). From Acute to Chronic: Unraveling the Pathophysiological Mechanisms of the Progression from Acute Kidney Injury to Acute Kidney Disease to Chronic Kidney Disease. Int. J. Mol. Sci..

[275] Lovisa S., Zeisberg M., Kalluri R. (2016). Partial Epithelial-to-Mesenchymal Transition and Other New Mechanisms of Kidney Fibrosis. Trends Endocrinol. Metab..

[276] La Russa A., Serra R., Faga T., Crugliano G., Bonelli A., Coppolino G., Bolignano D., Battaglia Y., Ielapi N., Costa D. (2024). Kidney Fibrosis and Matrix Metalloproteinases (Mmps). Front. Biosci.-Landmark.

[277] Evenepoel P., Poesen R., Meijers B. (2017). The Gut-Kidney Axis. Pediatr. Nephrol..

[278] Ramya Ranjan Nayak S.P., Boopathi S., Haridevamuthu B., Arockiaraj J. (2023). Toxic Ties: Unraveling the Complex Relationship between Endocrine Disrupting Chemicals and Chronic Kidney Disease. Environ. Pollut..

[279] Hayeeawaema F., Muangnil P., Jiangsakul J., Tipbunjong C., Huipao N., Khuituan P. (2023). A Novel Model of Adenine-Induced Chronic Kidney Disease-Associated Gastrointestinal Dysfunction in Mice: The Gut-Kidney Axis. Saudi J. Biol. Sci..

[280] Lu X., Ma J., Li R. (2023). Alterations of Gut Microbiota in Biopsy-Proven Diabetic Nephropathy and a Long History of Diabetes without Kidney Damage. Sci. Rep..

[281] Jiang S., Xie S., Lv D., Zhang Y., Deng J., Zeng L., Chen Y. (2016). A Reduction in the Butyrate Producing Species *Roseburia* Spp. and *Faecalibacterium prausnitzii* Is Associated with Chronic Kidney Disease Progression. Antonie Van Leeuwenhoek.

[282] Wong J., Piceno Y.M., DeSantis T.Z., Pahl M., Andersen G.L., Vaziri N.D. (2014). Expansion of Urease- and Uricase-Containing, Indole- and P-Cresol-Forming and Contraction of Short-Chain Fatty Acid-Producing Intestinal Microbiota in Esrd. Am. J. Nephrol..

[283] Marques F.Z., Nelson E., Chu P.Y., Horlock D., Fiedler A., Ziemann M., Tan J.K., Kuruppu S., Rajapakse N.W., El-Osta A. (2017). High-Fiber Diet and Acetate Supplementation Change the Gut Microbiota and Prevent the Development of Hypertension and Heart Failure in Hypertensive Mice. Circulation.

[284] Liang Z., Tang Z., Zhu C., Li F., Chen S., Han X., Zheng R., Hu X., Lin R., Pei Q. (2024). Intestinal Cxcr6+ Ilc3s Migrate to the Kidney and Exacerbate Renal Fibrosis Via Il-23 Receptor Signaling Enhanced by Pd-1 Expression. Immunity.

[285] Yan J., Herzog J.W., Tsang K., Brennan C.A., Bower M.A., Garrett W.S., Sartor B.R., Aliprantis A.O., Charles J.F. (2016). Gut Microbiota Induce Igf-1 and Promote Bone Formation and Growth. Proc. Natl. Acad. Sci. USA.

[286] Gleeson P.J., Benech N., Chemouny J., Metallinou E., Berthelot L., da Silva J., Bex-Coudrat J., Boedec E., Canesi F., Bounaix C. (2024). The Gut Microbiota Posttranslationally Modifies Iga1 in Autoimmune Glomerulonephritis. Sci. Transl. Med..

[287] Lan T., Tang T., Li Y., Duan Y., Yuan Q., Liu W., Ren Y., Li N., Liu X., Zhang Y. (2023). Ftz Polysaccharides Ameliorate Kidney Injury in Diabetic Mice by Regulating Gut-Kidney Axis. Phytomedicine.

[288] Kunter U., Seikrit C., Floege J. (2023). Novel Agents for Treating Iga Nephropathy. Curr. Opin. Nephrol. Hypertens..

[289] Zhang Z.-W., Han P., Fu J., Yu H., Xu H., Hu J.-C., Lu J.-Y., Yang X.-Y., Zhang H.-J., Bu M.-M. (2023). Gut microbiota-based metabolites of Xiaoyao Pills (a typical Traditional Chinese medicine) ameliorate depression by inhibiting fatty acid amide hydrolase levels in brain. J. Ethnopharmacol..

[290] Zhang Z.-W., Gao C.-S., Zhang H., Yang J., Wang Y.-P., Pan L.-B., Yu H., He C.-Y., Luo H.-B., Zhao Z.-X. (2022). Morinda officinalis oligosaccharides increase serotonin in the brain and ameliorate depression via promoting 5-hydroxytryptophan production in the gut microbiota. Acta Pharm. Sin. B.

[291] Wang Y., Tong Q., Ma S.-R., Zhao Z.-X., Pan L.-B., Cong L., Han P., Peng R., Yu H., Lin Y. (2021). Oral berberine improves brain dopa/dopamine levels to ameliorate Parkinson’s disease by regulating gut microbiota. Signal Transduct. Target. Ther..

[292] Feng R., Shou J.-W., Zhao Z.-X., He C.-Y., Ma C., Huang M., Fu J., Tan X.-S., Li X.-Y., Wen B.-Y. (2015). Transforming berberine into its intestine-absorbable form by the gut microbiota. Sci. Rep..

[293] Wu G.D., Chen J., Hoffmann C., Bittinger K., Chen Y.-Y., Keilbaugh S.A., Bewtra M., Knights D., Walters W.A., Knight R. (2011). Linking Long-Term Dietary Patterns with Gut Microbial Enterotypes. Science.

[294] Turnbaugh P.J., Ridaura V.K., Faith J.J., Rey F.E., Knight R., Gordon J.I. (2009). The Effect of Diet on the Human Gut Microbiome: A Metagenomic Analysis in Humanized Gnotobiotic Mice. Sci. Transl. Med..

