# Supplementary material for: Renal Health Through Medicine–Food Homology: A Comprehensive Review of Botanical Micronutrients and Their Mechanisms
Source: Nutrients. 2024 Oct 18;16(20):3530. doi: 10.3390/nu16203530 (PMC11510533; doi:10.3390/nu16203530)
Supplement: Supplementary file 1 [file nutrients-16-03530-s001.zip › nutrients-3208597-supplementary.pdf]

Table S1 Botanicals that are allowed to be added to dietary supplements in China.

Latin Name	EMA Herbal Medicine	EFSA Compendium	NIH DSLD
Panax ginseng C. A. Mey.	√	√	√
Panax notoginseng (Burkill) F. H. Chen ex C. H. Chow		√	√
Poria Cocos (Schw.) Wolf.			
Cirsium japonicum Fisch. ex DC.		√	
Ligustrum lucidum Ait.		√	
Cornus officinalis Sieb. et Zucc.		√	
Cyathula officinalis Kuan		√	
Fritillaria cirrhosa		√	
Ligusticum Chuanxiong			
Salvia miltiorrhiza Bunge	√	√	
Acanthopanar gracilistμlus W. W. Smith			
Schisandra chinensis (Turcz.) Baill.		√	
Actaea cimicifuga L.		√	
Asparagus cochinchinensis (Lour.) Merr.		√	
Gastrodia elata Bl.		√	√
Pseudostellaria heterophylla		√	√
Morinda officinalis		√	
Aucklandia lappa Decne.			
Equisetum hyemale L.		√	
Arctium lappa L.	√	√	
Plantago asiatica L.		√	
Glehnia littoralis F. Schmidt ex Miq.		√	√
Fritillaria ussuriensis Maxim.		√	
Scrophularia ningpoensis Hemsl.		√	√
Rehmannia glutinosa Libosch.		√	√
Pleuropterus multiflorus (Thunb.) Nakai			
Bletilla striata (Thunb. ex Murray) Rchb. F.			
Atractylodes macrocephala Koidz.		√	
Cynanchum otophyllum Schneid.			√
Amomum verum Blackw.		√	
Dendrobium nobile Lindl.			
Lycium barbarum L. (root)		√	√

Angelica sinensis (Oliv.) Diels	√	√	√
Phyllostachys nigra		√	
Carthamus tinctorius L.		√	
Rhodiola rosea L.	√	√	
Panax quinquefolius L.		√	√
Tetradium ruticarpum (A. Jussieu) T. G. Hartley		√	
Achyranthes bidentata Blume		√	
Eucommia ulmoides Oliv.		√	√
Paeonia suffruticosa		√	√
Aloe vera L.		√	√
Atractylodes Lancea Thunb. DC.		√	
Psoralea corylifolia Linn.		√	
Terminalia chebula Retz.		√	
Paeonia lactiflora pall / Paeonia veitchii lynch		√	
Polygala tenuifolia Willd.		√	
Ophiopogon japonicus (L. f.) Ker Gawl.		√	
Eupatorium fortunei Turcz.			
Platycladus orientalis		√	
Rheum palmatum L.	√	√	
Eleutherococcus senticosus (Rupr. & Maxim.) Maxim.	√	√	√
Rosa davurica Pall.			
Lycopus lucidus Turcz. var. *hirtus* Regel		√	
Alisma orientatle (Sam.) Juzep.		√	√
Rosa rugosa Thunb.		√	
Hibiscus sabdariffa L.		√	√
Anemarrhena asphodeloides Bunge		√	√
Apocynum venetum L.		√	√
Ilex latifolia Thunb.			
Fagopyrum dibotrys (D. Don) Hara			
Rosa laevigata Michx.		√	√
Citrus reticulata Blanco		√	√
Magnolia officinalis Rehd. et Wils.		√	
Curcuma longa L.	√	√	√
Citrus aurantium L.			
Platycladus orientalis (L.) Franco (seed)		√	

Gynostemma pentaphyllum (Thunb.) Makino		√	
Trigonella foenum-graecum L.	√	√	
Rubia cordifolia L.		√	
Piper longum L.		√	
Allium tuberosum Rottl. (seed)		√	√
Polygonum multiflorum Thunb.		√	
Cyperus rotundus L.		√	√
Davallia trichomanoides Blume			
Codonopsis pilosula (Franch.) Nannf.		√	√
Morus alba L.		√	√
Fritillaria thunbergii Miq.		√	
Leonurus japonicus Houtt.		√	
Centella asiatica	√	√	√
Epimedium brevicornu Maxim.			
Cuscuta chinensis Lam.		√	
Chrysanthemum indicum L.		√	
Ginkgo biloba L.	√	√	√
Astragalus membranaceus (Fisch.) Bge. var. *mongholicus* (Bge.) Hsiao/ *Astragalus membranaceus* (Fisch.) Bge.		√	√
Fritillaria hupehensis Hsiao et K.C.Hsia			
Cassia acufifolia			
Vaccinium vitis-idaea L.		√	
Sophora japonica L.		√	√
Typha angustifolia L.		√	
Tribulus terrestris L.	√	√	√
Tamarindus indica L.		√	
Eclipta prostrata L.		√	

EMA: European Medicines Agency; EFSA: European Food Safety Authority; NIH: National Institutes of Health; DSLD: Dietary Supplement Label Database; √: Indicating this botanical also appears in the index or database of ingredients; var.: Variety; f.: Form; cv.: Cultivar.
